# Supplementary material for: Chirality amplification by desymmetrization of chiral ligand-capped nanoparticles to nanorods quantified in soft condensed matter
Source: Nat Commun. 2018 Sep 25;9:3908. doi: 10.1038/s41467-018-06400-0 (PMC6156227; doi:10.1038/s41467-018-06400-0)
Supplement: Supplementary file 1 — Supplementary Information [file 41467_2018_6400_MOESM1_ESM.pdf]

Supplementary Information | **Chirality amplification by desymmetrization of chiral ligand-capped nanoparticles to nanorods quantified in soft condensed matter**

*Ahlam Nemati, Sasan Shadpour, Lara Querciagrossa, Lin Li, Taizo Mori, Min Gao, Claudio Zannoni and Torsten Hegmann**

*Correspondence to TH: thegmann@kent.edu

List of Supplementary Notes, Figures, and Appendices

Supplementary Note 1 | **Synthesis** ... 2

GNR synthesis ... 3

NMR spectra ... 4

Supplementary Note 2 | **Additional TEM images of the two GNRs** ... 7

Supplementary Note 3 | **Thermogravimetric analysis (TGA) of the GNRs** ... 8

Supplementary Note 4 | **Calculations** ... 9

Supplementary Note 5 | **Idealized *vs.* realistic ligand-shell coverage** ... 25

Supplementary Note 6 | **Thermal analysis (DSC)** ... 26

Supplementary Note 7 | **Handedness of induced N* LC phase** ... 28

Supplementary Note 8 | **Thin film ICD spectra of N* LC films** ... 29

Supplementary Note 9 | **Helical pitch measurements for GNRs** ... 30

I) Free surface (bottom substrate: glass, top substrate: air) ... 30

II) Grandjean-Cano wedge cells ... 33

Supplementary Note 10 | **Helical pitch measurements: COC and ZLI-4572 in 5CB** ... 38

Supplementary Note 11 | **Persistence length calculation (inter-particle distance)** ... 39

Supplementary Note 12 | **Felix-2900-03 – a different N-LC host** ... 40

Supplementary Note 13 | **Additional FF-TEM images** ... 41

Supplementary Note 14 | **Capped particles chirality quantification** ... 43

References ... 45

Appendix | **MATLAB code** ... 46

Supplementary Note 1 | **Synthesis**

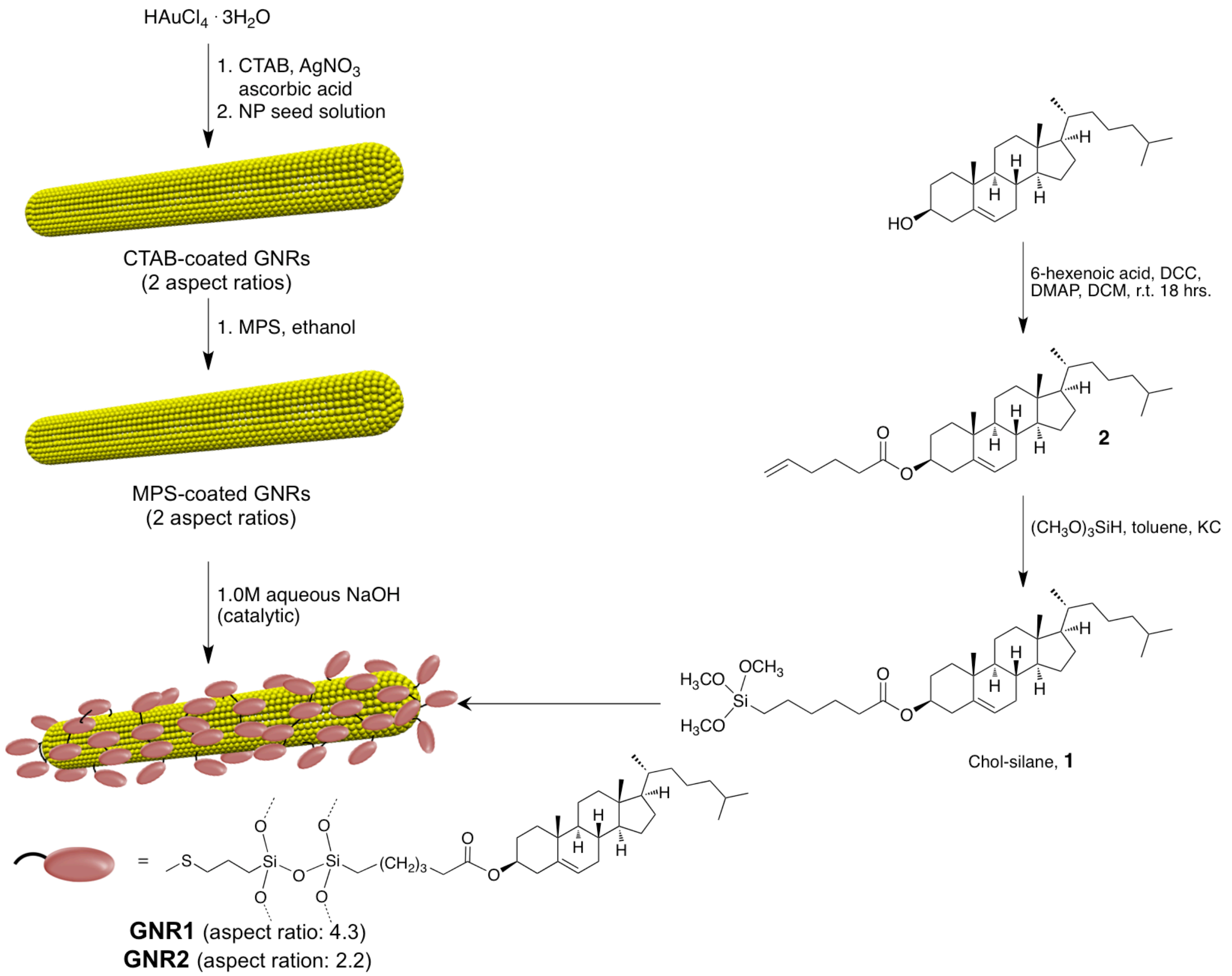

Supplementary Figure 1 | **Synthesis.** Synthetic pathway followed for the synthesis of GNRs (**GNR1** and **GNR2**) based on the siloxane-conjugation of Chol-silane **1** to MPS-coated GNRs. Abbreviations: MPS = (3-mercaptopropyl)trimethoxysilane, DCM = dichloromethane, KC = Karstedt's catalyst (platinum(0)-1,3-divinyl-1,1,3,3-tetramethyldisiloxane), DMAP = 4-(dimethylamino)pyridine, and CTAB = cetyltrimethylammonium bromide.

Ligand synthesis[1]

Compound **2**: Cholesterol (5.0 g, 25.9 mmol), 5-hexenoic acid (1.28 g, 31.1 mol), 4-(dimethyl-amino)pyridine (0.025 g, 0.40 mmol) and dicyclohexylcarbodiimide (3.2 g, 30.5 mmol) were added, and the mixture was stirred at room temperature for 20 hours. The dicyclohexylurea formed during the reaction was removed by filtration, and the filtrate was concentrated under reduced pressure. The filtrate was dissolved in hexane and any residue solid was discarded.

The precipitate was washed plentifully with ethanol. Finally, the crude product was purified by recrystallization by ethanol (and methanol) to give white solid and dried under vacuum at 40 °C. ^{1}H NMR ($CDCl_3$, 400 MHz, *δ*/ppm): 0.69 (s, 3H, CH_3), 0.85-2.17 (m, 43H, -CH_2-CH_2, CO_2-CH_2-CH_2, cholesteryl-CH, CH_2), 2.20-2.40 (m, 4H, C=C-CH_2-CH-O, CO_2-CH_2), 4.57- 4.66 (m, 1H, cholesteryl-O-CH), 4.96- 5.08 (m, 2H, -CH_2-CH=CH_2), 5.35- 5.41 (d, 1H, cholesteryl-C=CH, *J* = 4.8 Hz), 5.73- 5.85 (m, 1H, -CH_2-CH=CH_2).

Chol-silane **1**: Compound **1** (0.56 g, 2.07 mmol) was dissolved in dry toluene (25 mL). Trimethoxysilane (0.66 g, 5.44 mmol) was added, and the resulting solution was allowed to stir for 30 minutes at room temperature. Then, platinum(0)-1,3-divinyl-1,1,3,3-tetramethyldisiloxane (*aka* Karstedt's catalyst) in xylene (20 mg, 0.01 mmol) was added, and the reaction was allowed to stir overnight at room temperature. The excess of trimethoxysilane and solvent were removed under reduced pressure. The residue was dissolved in dichloromethane/ethyl acetate (1:1) and any undissolved part was discarded. The filtrate was collected, and the solvent removed under reduced pressure to obtain a sticky white-yellow solid. ^{1}H NMR ($CDCl_3$, 400 MHz, *δ*/ppm): 0.69 (s, 3H, CH_3), 0.68- 0.65 (m, 2H, -CH_2-Si-$(OCH_3)_3$), 0.85-2.17 (m, 47H, -CH_2-CH_2, CO_2-CH_2-CH_2, cholesteryl-CH, CH_2), 2.25-2.36 (m, 4H, C=C-CH_2-CH-O, CO_2-CH_2), 3.58 (s, 9H, -Si-$(OCH_3)_3$), 4.57- 4.68 (m, 1H, O-CH), 5.37- 5.41 (d, 1H, cholesteryl-C=CH, *J* = 4.6 Hz).

GNR synthesis

CTAB-coated GNRs: CTAB-coated GNRs were prepared following a slightly modified procedure first reported by El-Sayed and co-workers[2]. A growth solution was prepared by adding a 5 mL of 0.1M CTAB solution in DI water (pH = 5.5), 250 µL of 0.01M $HAuCl_4$, and 100 µL for **GNR2** (or 300 µL for **GNR1**) of a 0.01M aqueous $AgNO_3$ solution in this order followed by low speed magnetic stirring. Then, 100 µL of a 0.1M aqueous ascorbic acid solution was added, and the mixture became colorless. Thereafter, 13 µL of the seed solution was added to the growth mixture and then slowly mixed for a few seconds. The color of the solution turned purple after about 15 minutes. The mixture was left at 25 – 30 °C for 24 hours.

For the seed solution preparation, 500 µL of an aqueous 0.01 M solution of $HAuCl_4$ was added to an aqueous CTAB solution (10 mL, 0.1M). After an orange color appeared, 500 µL of an aqueous 0.01M ice-cold $NaBH_4$ solution was added. After 1 minute the solution developed a pale brown-yellow color. As outlined in Supplementary Table 1, by modifying the concentration of $AgNO_3$ and adjusting the pH, two aspect ratios of GNRs were made (**GNR1** and **GNR2**):

Supplementary Table 1 | **Synthesis parameters for the two aspect ratio GNRs.**

GNR	pH	Volume of 0.01M aqueous $AgNO_3$ solution added
**GNR1**	2.1	300 µL
**GNR2**	5.5	100 µL

Chol-silane (**1**)-capped **GNR1** and **GNR2**: Exchange of the CTAB bilayer on the GNR surface for the siloxane-conjugated MPS-chol-silane **1** was performed as described previously[3, 4, 5]. In a two-step approach, CTAB is first replaced by MPS followed by addition of Chol-silane **1** and adjustment of the pH to slightly basic conditions using 40 µL of aqueous 1M NaOH solution. The phase transfer from the aqueous to the organic layer in the used biphasic solvent system (H_2O-$CHCl_3$) can be observed visually – see Fig 2e.

NMR spectra

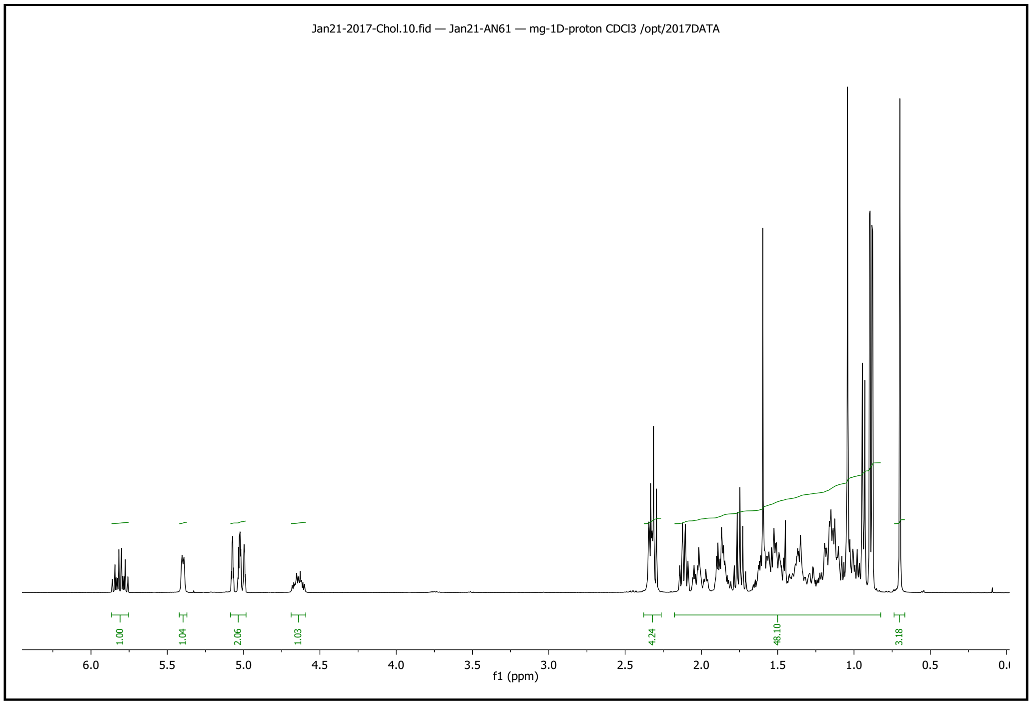

Supplementary Figure 2 | **NMR characterization.** ^{1}H NMR spectrum of compound **2**.

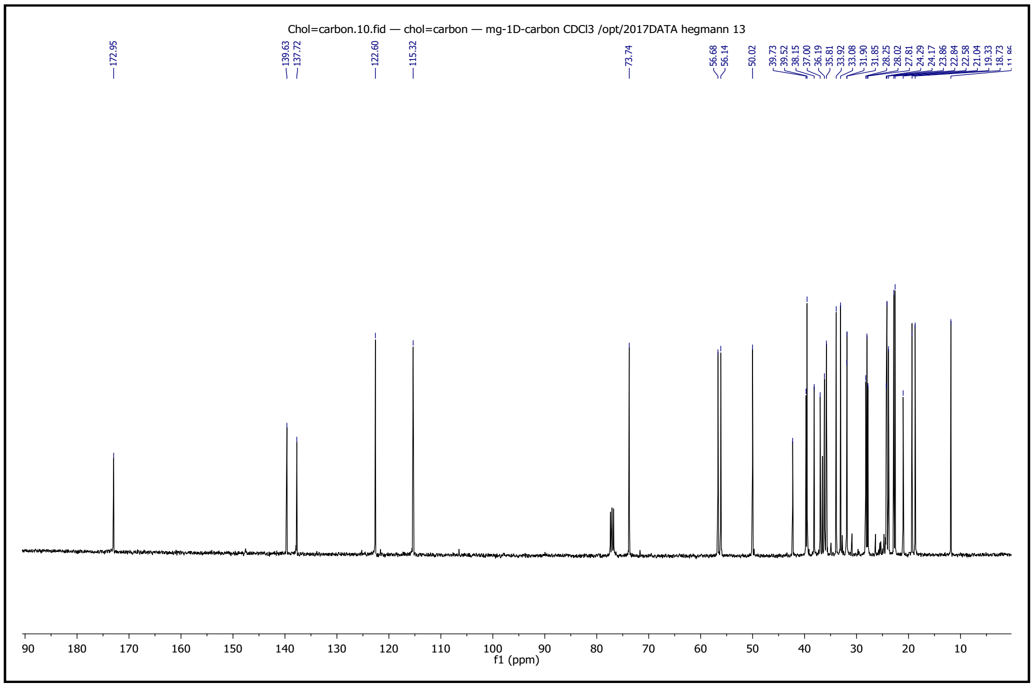

Supplementary Figure 3 | **NMR characterization.** ^{13}C NMR spectrum of compound **2**.

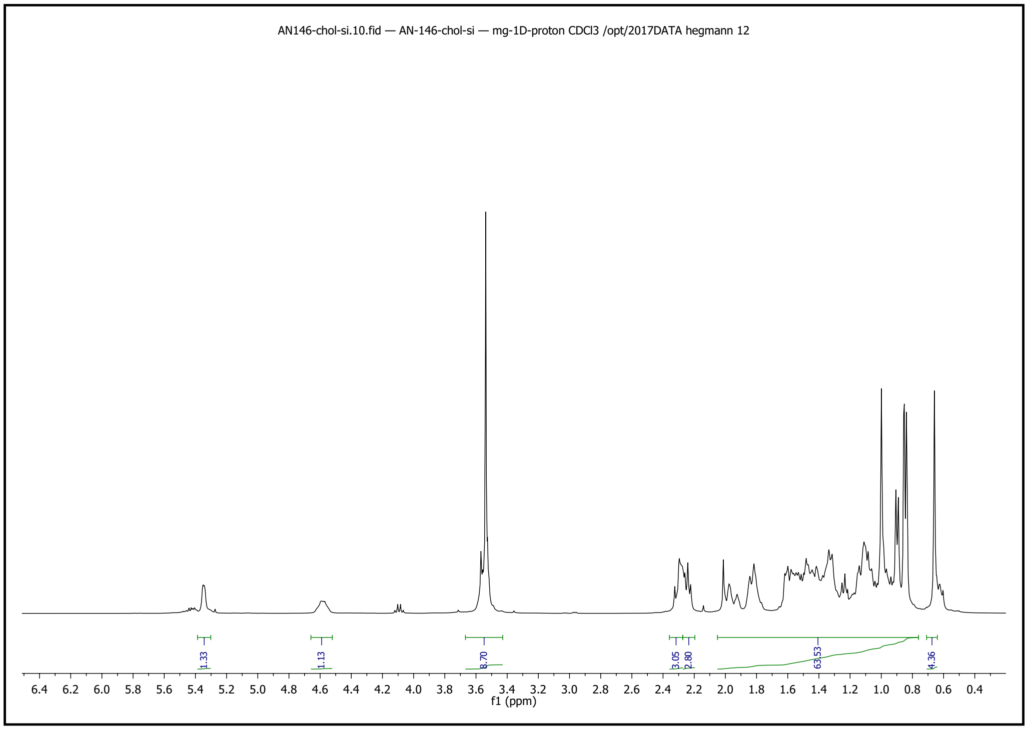

Supplementary Figure 4 | **NMR characterization.** ^{1}H NMR spectrum of Chol-silane **1**.

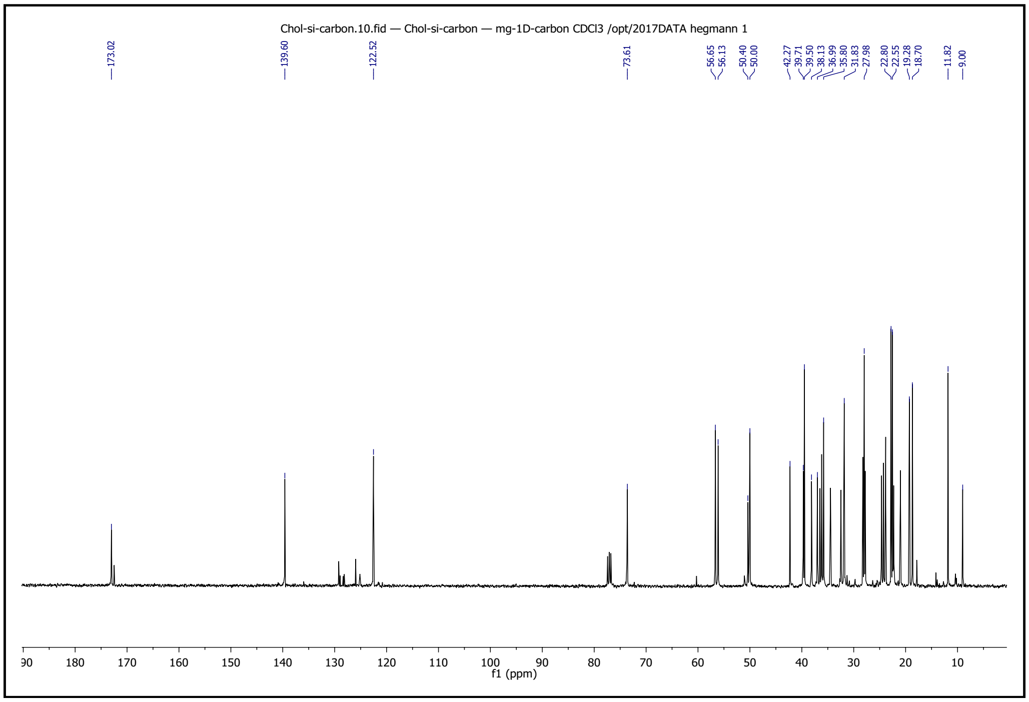

Supplementary Figure 5 | **NMR characterization.** ^{13}C NMR spectrum of Chol-silane **1**.

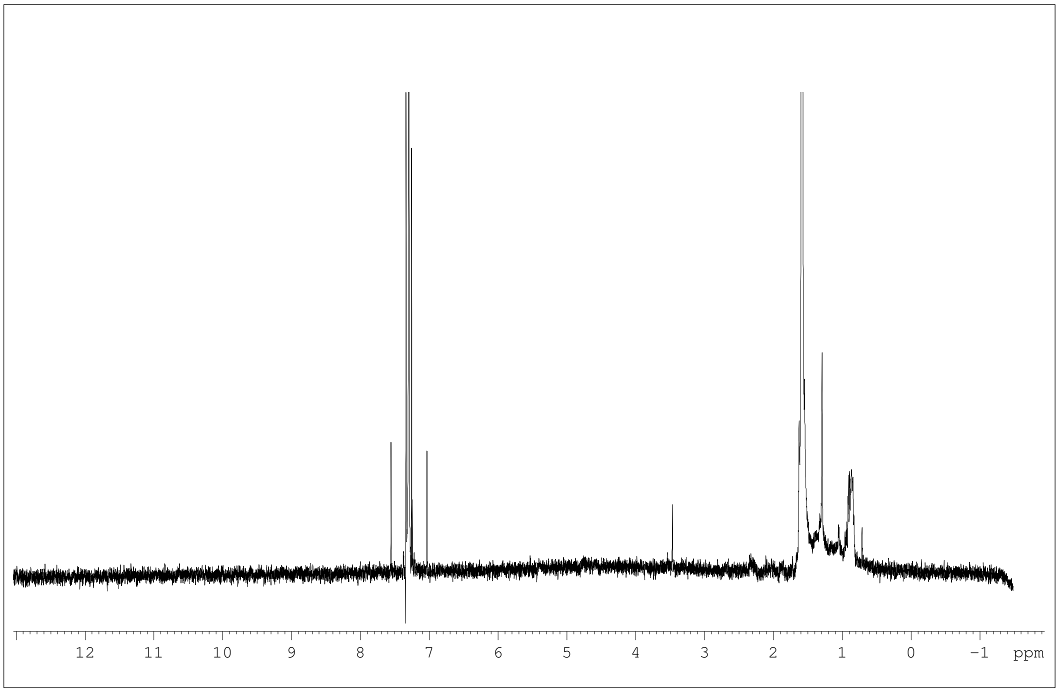

Supplementary Figure 6 | **NMR characterization.** ^{1}H NMR spectrum of Chol-silane **1** functionalized **GNR1** (representatively for both **GNR1** and **GNR2**, only differing in aspect ratio). Typical for gold nanostructures with ligand shells, only broad peaks are observed indicating the absence of free, non-bound ligands[6].

Supplementary Note 2 | **Additional TEM images of the two GNRs**

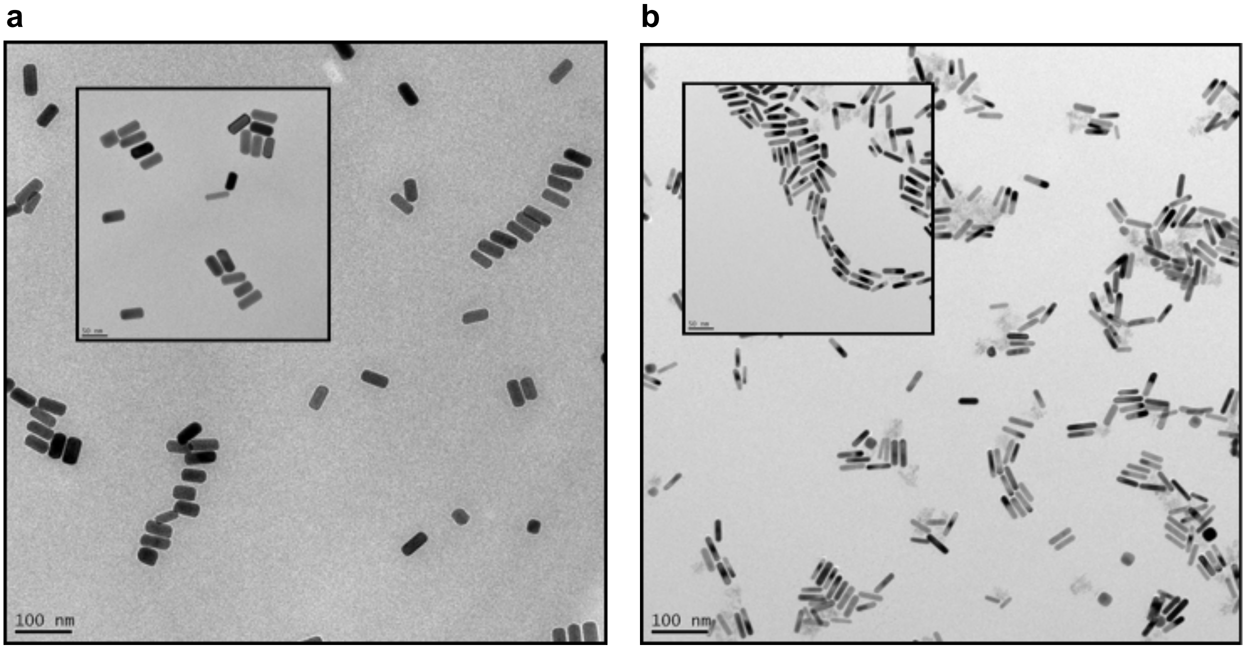

Supplementary Figure 7 | **Additional TEM images.** TEM images of: **a, GNR2** with smaller aspect ratio and **b, GNR1** with larger aspect ratio (scale bar in each inset = 50 nm).

Supplementary Note 3 | **Thermogravimetric analysis (TGA) of the GNRs**

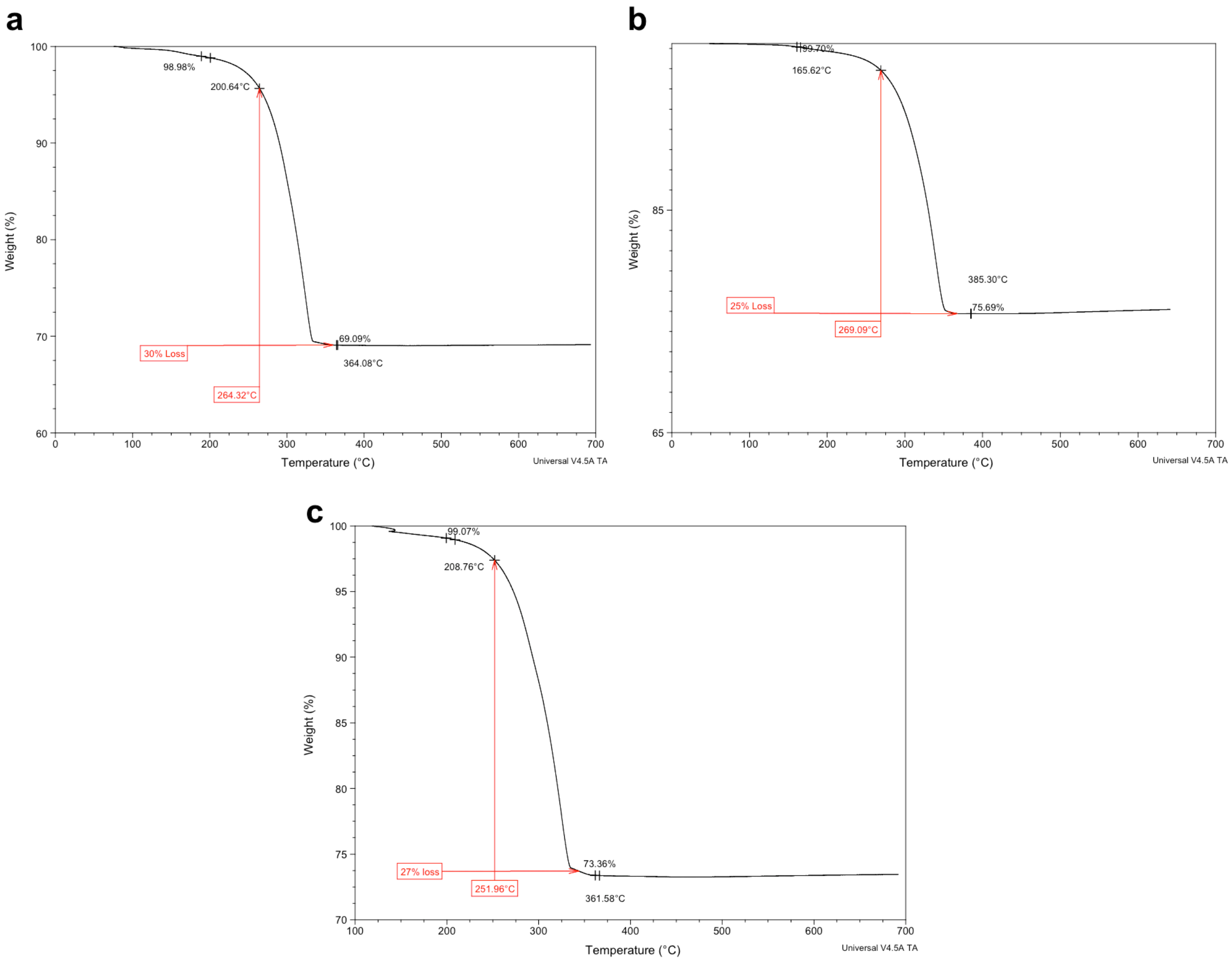

Supplementary Figure 8 | **Determination of wt.% of ligand shell.** Thermogravimetric analysis (TGA) plots for the two GNR samples: **a GNR1** (using a 12 mg sample – weight loss: 30 % - identical for multiple samples) and **b GNR2** (using a 20 mg sample – weight loss: 25%), and **c GNR2** (using a second 10 mg sample – weight loss: 27%). We used the average for **GNR2**: 26%.

Supplementary Note 4 | **Calculations**

Method 1

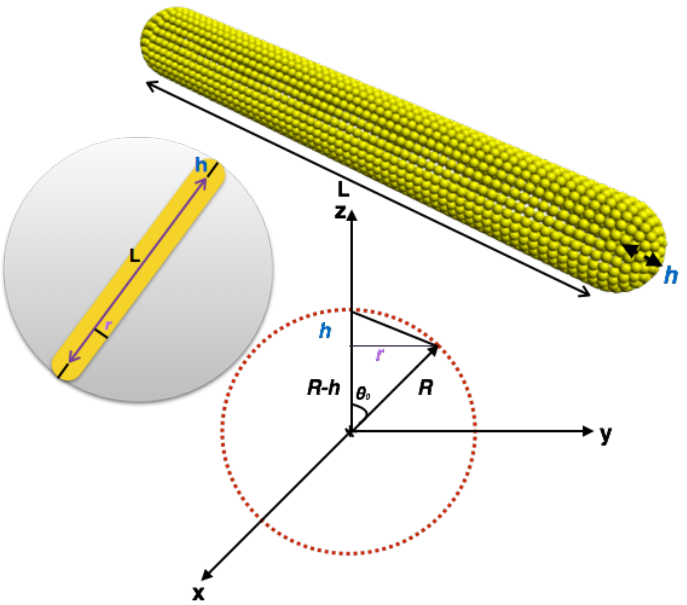

Supplementary Figure 9 | **Model used for composition calculation.** Schematic illustration of the model and parameters for calculation of the GNRs' surface area used in Method 1.

$$A_1 = 2\pi r l$$

$$A_2 = \int R^2 \sin\theta d\theta d\varphi = -R^2 cos\theta \begin{pmatrix} \theta = \theta_0 \\ \theta = 0 \end{pmatrix}$$

$$2\pi R^2(1 - cos\theta_0) \quad , \quad tan\theta_0 = \frac{r}{R-h}$$

$$1 + tan^2 = 1 + \frac{sin^2\theta}{\cos^2\theta} = \frac{1}{\cos^2\theta} \qquad \cos\theta = \frac{1}{\sqrt{1+\tan^2\theta}}$$

$$\cos\theta_0 = \frac{1}{\sqrt{1+\left(\frac{r}{R-h}\right)^2}} \quad , \quad A_2 = 2\pi R^2\left(1 - \frac{1}{\sqrt{1+\left(\frac{r}{R-h}\right)^2}}\right)$$

$$r^2 + (R-h)^2 = R^2 \qquad r^2 + R^2 - 2Rh + h^2 = R^2 \qquad R = \frac{r^2+h^2}{2h}$$

$$A = A_1 + 2A_2$$

All GNR parameters (r, h, L) were calculated using ImageJ[7]. Data are shown in Supplementary Tables 2 and 3.

GNR1:

Average sized from TEM (nm): $L\ (\mathbf{42.73\ nm}) \times W\ (\mathbf{10.15}\ nm)$

Average aspect ratio (*L*/*W*): 4.25

Length of cylinder (nm): $L = 42.73 - 10.15 = 32.58\ (nm)$

Radius of hemisphere (nm):

$$r = \frac{W}{2} = \frac{10.15}{2} = 5.075\ n\text{m}$$

Supplementary Table 2 | **Average dimensions of GNR1 (larger aspect ratio).**

#	Length / nm	Width / nm	Angle / °	Area / nm^2	Aspect Ratio	*h* / nm
1	39.17	9.68	-18.43	1.79	4.04	3.20
2	41.49	11.10	-105.94	2.05	3.73	3.44
3	39.96	8.81	-11.31	1.54	4.53	2.64
4	44.78	8.78	146.31	1.02	5.09	1.70
5	44.16	8.99	-135.00	1.54	4.91	2.50
6	38.76	7.93	180.00	1.79	4.88	3.04
7	44.45	9.27	8.13	2.05	4.79	3.30
8	38.80	9.26	0.00	1.54	3.75	2.53
9	42.15	9.86	-123.69	1.28	4.27	1.97
10	42.32	8.32	180.00	1.54	5.08	2.53
11	43.94	10.00	-135.00	2.05	4.39	3.76
12	37.65	7.89	-63.43	1.54	4.76	2.49
13	34.79	8.44	-45.00	1.28	4.12	2.07
14	40.46	6.65	-56.31	1.28	4.57	1.87
15	39.32	10.43	-51.34	1.79	3.76	2.99
16	39.60	8.51	-50.19	2.05	4.65	3.77
17	39.67	10.99	-53.13	1.79	3.60	2.99
18	47.85	10.92	-45.00	2.56	4.38	4.39
19	44.36	13.67	-45.00	1.79	3.24	2.95
20	43.57	11.43	-45.00	1.28	3.81	2.27

21	39.23	8.35	-30.96	1.54	4.69	2.72
22	42.50	10.07	-45.00	1.54	4.21	2.63
23	47.53	11.04	0.00	1.79	4.30	3.12
24	49.90	10.36	-45.00	1.79	4.81	3.10
25	42.46	9.83	-30.96	1.79	4.31	2.97
26	38.09	10.75	0.00	1.79	3.54	2.79
27	41.70	8.74	0.00	1.28	3.62	1.94
28	36.35	8.98	-30.96	1.54	4.04	2.75
29	44.93	11.92	23.19	2.31	3.76	3.89
30	26.31	8.08	9.46	1.79	3.25	3.26
31	40.84	9.06	-161.56	1.79	4.50	2.91
32	43.05	11.84	-53.13	1.54	3.63	2.43
33	48.65	10.13	18.43	2.05	4.80	3.40
34	43.11	9.98	23.19	2.05	4.31	3.45
35	43.14	8.50	-165.96	1.54	5.07	2.29
36	39.39	9.73	26.56	2.56	4.04	4.38
37	40.60	11.55	90.00	1.54	3.51	2.63
38	39.22	9.70	-101.31	1.54	4.04	2.48
39	40.82	9.57	-45.00	1.79	4.26	3.10
40	43.96	12.21	-30.96	1.79	3.59	2.97
41	42.37	12.07	0.00	1.79	3.51	2.79
42	43.71	11.32	18.43	2.05	3.86	3.40
43	40.50	9.20	23.19	2.05	4.40	3.45
44	42.11	9.23	-165.96	1.54	4.55	2.29
45	45.64	10.89	26.56	2.56	4.19	4.38
46	40.63	10.61	90.00	1.54	4.77	2.63
47	42.89	8.12	-101.31	1.54	5.28	2.48
48	44.71	11.78	180.00	1.79	3.96	3.04
49	42.98	9.16	8.13	2.05	4.68	3.30
50	38.62	8.95	0.00	1.54	4.31	2.53
51	47.57	11.77	-123.69	1.28	4.04	1.97
52	42.11	11.10	180.00	1.54	3.79	2.53
53	45.33	11.55	-135.00	2.05	4.09	3.76

54	46.45	10.44	-63.43	1.541	4.44	2.49
55	39.60	9.15	-45.00	1.284	4.32	2.07
56	49.31	11.32	-56.31	1.28	4.35	1.87
57	42.90	12.36	-51.34	1.79	3.47	2.99
58	39.62	9.06	-50.19	2.05	3.93	3.77
59	45.49	9.98	-53.13	1.79	4.55	2.99
60	46.30	10.15	-45.00	2.56	4.56	4.39
61	46.29	10.25	-45.00	1.79	4.51	2.95
62	43.58	8.98	-45.00	1.28	4.85	2.27
63	45.65	11.76	-30.96	1.541	4.22	2.72
64	45.52	12.53	-45.00	1.541	3.63	2.63
65	43.62	9.47	0.00	1.79	4.60	3.12
66	43.02	8.18	-45.00	1.79	5.25	3.10
67	47.00	10.75	-30.96	1.79	4.37	2.97
68	39.06	9.46	0.00	1.79	4.12	2.79
69	42.56	10.98	0.00	1.28	3.87	1.94
70	43.42	9.79	-30.96	1.54	4.43	2.75
71	42.50	11.04	23.19	2.31	3.85	3.89
72	38.76	9.55	9.46	1.79	4.05	3.26
73	43.99	11.67	-161.56	1.79	3.76	2.91
74	42.49	11.21	-53.13	1.54	3.79	2.43
75	39.34	8.81	18.43	2.05	4.46	3.40
76	43.52	9.36	23.19	2.05	6.14	3.45
77	41.24	8.59	-59.03	1.79	3.63	2.91
78	44.77	11.77	74.05	2.05	3.80	3.56
79	42.10	9.38	90.00	2.31	4.48	4.05
80	45.13	11.64	-56.31	2.05	3.87	3.52
81	44.17	11.40	90.00	2.05	3.87	3.67
82	48.89	11.23	0.00	2.05	4.35	3.42
83	44.49	8.87	-30.96	1.79	5.01	3.09
84	42.50	8.15	0.00	1.79	5.21	3.04
85	40.79	10.86	-45.00	2.05	4.67	3.58
86	40.26	10.36	-45.00	1.79	3.88	2.95

87	43.93	9.43	68.19	1.79	4.65	3.17
88	43.02	12.30	180.00	1.79	3.49	2.91
89	45.45	10.54	56.31	1.28	4.31	1.87
90	39.88	10.25	-45.00	1.79	3.88	2.86
91	48.74	11.66	-45.00	1.79	4.17	2.86
92	44.03	8.80	-116.56	1.54	5.00	2.28
93	45.31	10.23	-126.87	1.54	4.43	2.39
94	45.84	11.01	-128.66	1.79	4.16	3.12
95	42.86	8.35	-126.87	1.54	5.13	2.74
96	42.41	10.58	-120.96	1.54	4.00	2.72
97	42.26	11.50	-120.96	1.78	3.67	3.06
98	43.92	11.32	-161.56	2.05	3.87	3.53
99	43.21	11.35	170.53	1.79	3.80	3.17
100	44.59	11.21	50.19	2.31	4.06	4.05
**AVG**	**42.73 ± 3.3**	**10.14 ± 1.3**			**4.25**	**2.98**

Number of Au atoms in one GNR:

$A_1 = 2\pi rl$ $\quad\quad A_2 = 2\pi R^2\left(1 - \frac{1}{\sqrt{1+\left(\frac{r}{R-h}\right)^2}}\right)$

Surface area: $A = A_1 + 2A_2 = 449.7\ nm^2$

$N_{Au} = \frac{\mathrm{A}}{A_{Au}} = 25953$

Number of ligands on the surface:

Surface area of thiol on gold atom = 21.4 Å

$N_{lig} = \frac{\mathrm{A}}{0.214} = 2141$

M_w of Au atoms in GNRs (g mol^{-1}):

$M_w(Au\ in\ GNR) = N_{Au} \times M_w(Au)$

$M_w(Au\ in\ GNR) = 5111850.6$ g mol^{-1}

M_w of Ligands in GNRs (g mol^{-1}):

$M_w(ligand\ in\ GNRs) = N_{lig} \times M_w(ligand)$

$M_w(ligand\ in\ GNRs) = 1717313.76$ g mol^{-1}

M_w of GNR + Ligands (g mol^{-1}):

$M_w(GNR + L) = M_w(Au\ in\ NR) + M_w(ligand\ in\ GNRs)$

$M_w(GNRs + L) = 6729164.36$ g mol^{-1}

Weight percentage of ligands in one GNR:

$$wt\%\ (ligand\ in\ GNRs) = \frac{M_w(ligand\ in\ GNRs)}{M_w(GNRs)} \times 100\%$$

$$wt\%\ (ligand\ in\ GNRs) = \frac{1717313}{6729164} \times 100\% = 26\%$$

GNR2:

Average size from TEM (nm): $L\ (51.27\ nm) \times W\ (23.48\ nm)$

Average aspect ratio (*L*/*W*): 2.21

Length of cylinder (nm): $L = 51.27 - 23.48 = 27.78\ (nm)$

Radius of hemisphere (nm):

$$r = \frac{W}{2} = \frac{23.48}{2} = 11.741\ nm$$

Supplementary Table 3 | **Average dimensions of GNR2 (smaller aspect ratio).**

#	Length / nm	Width / nm	Angle / °	Area / nm^2	Aspect Ratio	*h* / nm
1	53.94	23.89	36.01	1.59	2.34	1.78
2	45.97	22.85	31.20	1.32	2.01	2.81
3	57.34	22.61	65.28	1.32	2.53	2.26
4	42.27	20.04	11.33	1.59	2.10	1.89
5	47.68	25.24	52.67	1.59	1.88	2.74
6	51.52	23.33	74.07	2.12	2.20	2.58
7	50.53	19.39	38.89	2.12	2.60	3.60
8	49.04	20.22	36.10	1.59	2.42	3.37
9	44.07	22.24	58.44	1.59	1.98	2.62

10	47.87	23.78	53.22	1.06	2.01	2.62
11	49.14	28.03	57.44	1.06	1.75	1.75
12	54.11	19.49	59.67	2.12	2.77	1.72
13	49.33	21.83	7.65	2.12	2.26	3.47
14	50.78	18.31	17.78	2.12	2.22	3.54
15	46.91	23.58	31.98	2.12	1.98	3.64
16	41.50	23.94	4.78	1.06	1.73	3.80
17	46.48	23.40	50.11	1.86	1.98	1.71
18	57.79	21.22	31.33	1.32	2.72	2.88
19	54.38	21.19	55.00	1.06	2.56	2.23
20	48.34	22.39	60.84	1.32	2.15	1.70
21	49.31	23.98	52.53	1.86	2.05	2.01
22	41.00	21.53	52.98	1.59	1.90	3.24
23	44.27	22.46	64.33	1.32	1.97	2.77
24	52.83	21.27	75.67	1.86	2.48	1.95
25	51.55	21.89	57.13	1.32	2.35	3.09
26	46.07	17.64	74.47	1.86	2.61	2.15
27	42.31	22.76	67.37	1.32	1.85	2.84
28	46.44	23.60	38.33	1.59	1.96	2.09
29	49.55	21.09	13.00	1.86	2.34	2.83
30	53.25	21.04	28.99	1.06	2.53	2.88
31	55.78	20.35	53.00	1.32	2.88	1.69
32	47.42	20.25	53.31	1.86	2.29	2.00
33	51.78	19.20	64.44	1.86	2.69	3.24
34	53.26	20.85	53.33	1.06	2.55	3.05
35	49.41	22.52	48.75	1.59	2.19	1.75
36	52.46	20.28	53.28	1.06	2.58	2.41
37	52.32	21.50	36.01	1.59	2.43	1.78
38	50.66	20.47	31.20	1.32	2.47	2.81
39	43.94	19.69	65.28	1.32	2.48	2.26
40	49.56	18.88	11.33	1.59	2.62	1.89
41	51.28	21.44	52.67	1.59	2.39	2.74
42	41.40	22.45	74.07	2.12	1.84	2.58

43	54.59	21.75	38.89	2.12	2.50	3.60
44	52.50	21.63	36.10	1.59	2.42	3.37
45	48.40	20.59	58.44	1.59	2.35	2.62
46	49.83	23.79	53.22	1.06	2.09	2.62
47	58.58	26.09	57.44	1.06	2.24	1.75
48	54.23	27.76	59.67	2.12	1.95	1.72
49	51.83	28.26	7.65	2.12	1.83	3.47
50	53.84	21.37	17.78	2.12	2.51	3.54
51	50.56	25.16	31.98	2.12	2.00	3.64
52	53.32	25.65	4.78	1.06	2.07	3.80
53	49.83	23.79	50.11	1.86	2.09	1.71
54	50.37	24.83	31.33	1.32	2.02	2.88
55	47.92	20.07	55.00	1.06	2.38	2.23
56	56.05	28.95	60.84	1.32	1.93	1.70
57	62.78	25.16	52.53	1.86	2.49	2.01
58	63.14	27.74	52.98	1.59	2.27	3.24
59	53.75	26.09	64.33	1.32	2.06	2.77
60	54.03	26.98	75.67	1.86	2.00	1.95
61	59.66	26.89	57.13	1.32	2.21	3.09
62	52.05	27.34	74.41	1.86	1.90	2.15
63	53.96	24.34	67.37	1.32	1.57	2.84
64	55.48	29.22	38.33	1.59	1.96	2.09
65	52.62	26.36	13.00	1.86	1.99	2.83
66	46.71	22.60	28.94	1.06	2.06	2.88
67	50.37	26.70	53.00	1.32	1.88	1.69
68	40.36	23.87	53.31	1.86	1.69	2.00
69	52.04	26.46	64.44	1.86	2.23	3.24
70	51.16	23.59	53.33	1.06	2.16	3.05
71	57.75	23.98	48.75	1.59	2.40	1.75
72	54.91	22.88	53.28	1.59	2.83	2.41
73	53.79	21.89	53.28	1.06	2.45	2.41
74	46.88	26.09	36.01	1.59	1.79	1.78
75	48.17	23.22	31.20	1.32	2.07	2.81

76	50.29	21.12	65.20	1.32	2.38	2.26
77	45.12	24.00	11.33	1.59	1.88	1.89
78	56.27	25.96	52.66	1.59	2.01	2.74
79	56.68	23.22	74.06	2.12	1.94	2.58
80	53.52	23.41	38.89	2.12	2.28	3.60
81	48.27	26.34	36.10	1.59	1.83	3.37
82	48.20	22.63	58.44	1.59	2.12	2.62
83	54.35	25.73	53.22	1.06	2.15	2.62
84	48.91	27.96	57.44	1.06	1.74	1.75
85	54.91	24.26	59.67	2.12	2.26	1.72
86	52.97	22.92	7.55	2.12	1.96	3.47
87	54.77	25.73	17.58	2.12	2.28	3.54
88	52.77	23.18	31.68	2.12	2.53	3.64
89	47.18	21.44	4.77	1.06	2.20	3.80
90	49.37	22.37	50.11	1.86	2.20	1.71
91	48.76	21.05	31.33	1.32	2.22	2.88
92	46.14	20.59	55.00	1.06	2.24	2.23
93	51.16	27.02	60.86	1.32	1.89	1.70
94	50.87	21.12	52.58	1.86	2.40	2.01
95	53.72	26.85	52.98	1.59	2.00	3.24
96	53.66	24.05	64.33	1.32	2.48	2.77
97	53.62	19.42	75.66	1.86	2.91	1.95
98	52.78	25.36	57.13	1.32	2.20	3.09
99	53.66	22.60	74.41	1.86	2.41	2.15
100	52.20	24.20	67.37	1.79	2.02	2.84
**AVG**	**50.96 ± 4.4**	**23.26 ± 2.4**			**2.21**	**2.59**

Number of Au atoms in one GNR:

$A_1 = 2\pi r l$ $\qquad$ $A_2 = 2\pi R^2 \left(1 - \frac{1}{\sqrt{1 + \left(\frac{r}{R-h}\right)^2}}\right)$

Surface area: $A = A_1 + 2A_2 = 3557.42$

$$N_{Au} = \frac{\mathrm{A}}{A_{Au}} = 209235$$

Number of ligands on the surface:

Surface area of thiol on gold atom = 21.4 Å

$$N_{lig} = \frac{\mathrm{A}}{0.214} = 16938$$

M_w of Au atoms in GNR (g mol^{-1})

$M_w(Au\ in\ GNRs) = N_{Au} \times M_w(Au)$

$M_w(Au\ in\ GNRs) = 41210925$ g mol^{-1}

M_w of Ligands (g mol^{-1}):

$M_w(ligand\ in\ GNRs) = N_{lig} \times M_w(ligand)$

$M_w(ligand\ in\ GNRs) = 12793062$ g mol^{-1}

M_w of GNR with Ligands (g mol^{-1}):

$M_w(GNRs + L) = M_w(Au\ in\ GNR) + M_w(ligand\ in\ GNR)$

$M_w(GNRs) = 41210925 + 12793062 = 54003987.14$ g mol^{-1}

Weight percentage of ligands in one GNR:

$$wt.\%\ (ligand\ in\ GNRs) = \frac{M_w(ligand\ in\ GNRs)}{M_w(GNRs)} \times 100\%$$

$$wt.\%\ (ligand\ in\ GNRs) = \frac{\mathbf{12793062}}{\mathbf{54003987.14}} \times 100\% = 24\%$$

Supplementary Table 4 | **Calculated composition data – method A.** Average number of gold atoms in the two GNRs and at the surface of the GNRs, and average weight percent of ligand shell on the GNR surface compared to experimental TGA data. Abbreviations: AR = aspect ratio, TGA = thermogravimetric analysis.

GNR	Length / nm	Width / nm	AR	N_{Au}	$N_{Chol\text{-}Silane}$	Wt.% ligands in GNR (calc.)	TGA / wt.%
**GNR1**	42.73	10.15	4.25	2.5×10^4	2.1×10^3	26	30
**GNR2**	51.32	23.48	2.21	2.0×10^5	1.6×10^4	24	26

Supplementary Table 5 | **Concentration (molarity) of chiral ligands and GNRs in 0.5 and 1 wt.% mixtures with 5CB.**

GNR	Length / nm	Widths / nm	Molar conc. of chiral ligand / M	Molar conc. of GNRs in 0.5 wt.% / M	Molar conc. of GNRs in 1 wt.%
**GNR1**	42.73	10.15	1.8×10^{-7}	7.4×10^{-7}	1.5×10^{-7}
**GNR2**	51.32	23.48	1.6×10^{-6}	9.2×10^{-7}	1.8×10^{-7}

Supplementary Table 6 | **Concentration (molarity) of chiral ligands and related Au NPs in 0.5 and 5 wt.% mixtures with 5CB[1].**

NP	Size / nm	Molar conc. of chiral ligand / M	Molar conc. of Au NPs in 0.5 wt.% / M	Molar conc. of Au NPs in 5 wt.% / M
**NP1**	1.77	1.7×10^{-4}	9.1×10^{-4}	9.4×10^{-3}
**NP2**	5.54	7.5×10^{-6}	1.9×10^{-5}	22.6×10^{-4}
**NP3**	10.09	1.3×10^{-6}	4.1×10^{-6}	3.0×10^{-5}

Supplementary Table 7 | **Number of Au NPs and chiral ligands for the related cholesterol-capped Au NPs at 0.5 and 5 wt.% in 5CB[1].**

NP	Size / nm	M_W Au NP + chiral ligands	Number of Au NP in LC mixture at 0.5 wt.%	Number of Au NP in LC mixture at 5 wt.%
**NP1**	1.77	5.7×10^{4}	5.3×10^{16}	5.5×10^{17}
**NP2**	5.54	1.2×10^{6}	2.3×10^{15}	2.4×10^{16}
**NP3**	10.09	7.3×10^{6}	4.1×10^{14}	5.3×10^{15}

Supplementary Table 8 | **Number of GNRs and chiral ligands at concentrations of 0.5 and 1 wt.%. in 5CB.**

GNR	Length / nm	Widths / nm	M_W of GNR-chiral ligand	Number of GNRs in LC at 0.5 wt.%	Number of GNRs in LC at 1 wt.%
**GNR1**	42.73	10.15	6.7×10^{6}	4.4×10^{15}	8.9×10^{15}
**GNR2**	51.32	23.48	5.4×10^{7}	1.1×10^{14}	5.5×10^{15}

Supplementary Table 9 | **Wt.% of chiral ligands on Au NPs, helical pitch *p*, and helical twisting power β_W of chiral ligands on Au NP surface (in 5CB)[1].**

NP	Size / nm	Wt.% of chiral ligands on Au NP	*p* of N* LC / µm at 5wt.%	β_W of chiral ligand on Au NP / μm^{-1} wt.%$^{-1}$
**NP1**	1.77	41.4	6.23 ± 0.5	−8.7
**NP2**	5.54	20.4	5.33 ± 0.69	−29.0
**NP3**	10.09	15.0	—[a]	—[a]

[a] Helical pitch was too large and could not be measured.

Supplementary Table 10 | **Concentration (wt.%) of chiral ligands on GNRs, helical pitch *p*, and helical twisting power β_W of chiral ligands on GNR surface (in 5CB).**

GNR	Dimensions / nm	Wt.% of chiral ligands on GNR	*p* of N* LC / µm at 0.5 wt.%	β_W of chiral ligand on GNR / μm^{-1} wt.%$^{-1}$
**GNR1**	42.73 × 10.15	26	2.4 ± 0.79	−382.0
**GNR2**	51.32 × 23.48	24	3.4 ± 0.71	−248.0

Supplementary Table 11 | **Number of chiral ligands in N*-LC at 0.5 and 5 wt.% used for the synthesis of the Au NPs in 5CB[1].**

Number of chiral ligands (for quasi-spherical Au NPs) in LC mixture per cm^3		
**Ligand**	**0.5 wt.% LC Mixture**	**5 wt.% LC mixture**
**Chol-disulfide 1**	2.17×10^{16}	2.2×10^{17}
**Chol-disulfide 2**	4.6×10^{14}	4.9×10^{16}
**Chol-silane 1**	6.1×10^{13}	1.1×10^{15}

Supplementary Table 12 | **Number of chiral ligands in N*-LC for 0.5 and 1 wt.% of the two GNRs in 5CB.**

Number of chiral ligands for GNRs in 5CB per cm^3	
**0.5 wt.%**	**1 wt.%**
**GNR1**	
1.1×10^{13}	2.1×10^{13}

GNR2	
1.2×10^{12}	2.6×10^{12}

Method 2

The second calculation model is based on the surface area of GNRs assuming a round cylinder and two hemispheres. The MATLAB code used for these calculations is provided Appendix 1 at the end of this document.

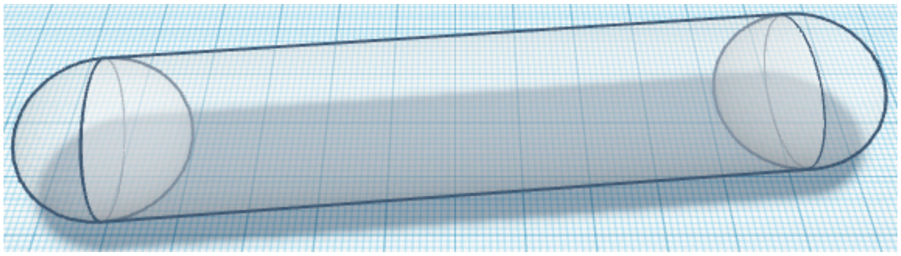

Supplementary Figure 10 | **Model used for MATLAB composition and aspect ratio calculation.** Schematic illustration showing Method 2 for calculation of the GNRs' surface area.

GNR1:

Average sized from TEM (nm): $L\ (\mathbf{50.02}\ nm) \times W\ (\mathbf{12.08}\ nm)$

Average aspect ratio (*L*/*W*): 4.16

Radius of Au core from TEM: 60 Å

Number of gold atoms at interface: 9613

Number of ligands on the surface:

Surface area of thiol on gold atom = 21.4 Å

$$N_{lig} = \frac{\mathrm{A}}{0.214} = 986$$

M_w of Au atoms in GNR (g mol^{-1})

$M_w(Au\ in\ GNRs) = N_{Au} \times M_w(Au)$= 1893431.63 g mol^{-1}

M_w of ligands in GNRs (g mol^{-1}):

$M_w(ligand\ in\ GNRs) = N_{lig} \times M_w(ligand)$ = 745078.34 g mol^{-1}

M_w of GNR + Ligand (g mol^{-1}):

$M_w(GNRs + L) = M_w(Au\ in\ GNR) + M_w(ligand\ in\ GNRs) = 2638509.79$ g mol^{-1}

Weight percentage of ligands in one GNR:

$wt\%\ (ligand\ in\ GNRs) = \frac{M_w(ligand\ in\ GNRs)}{M_w(GNRs)} \times 100\% = 28\%$

GNR2:

Average sized from TEM (nm): $L\ (\mathbf{50.03}\ nm) \times W\ (\mathbf{19.95}\ nm)$

Average aspect ratio (*L*/*W*): 2.5

Radius of Au core from TEM: 100 Å

Number of gold atoms at interface: 15700

Number of ligands on the surface:

Surface area of thiol on gold atom = 21.4 Å

$N_{lig} = \frac{\mathrm{A}}{0.214} = 1760$

M_w of Au atoms in GNRs (g mol^{-1})

$M_w(Au\ in\ GNR) = N_{Au} \times M_w(Au)$= 3092272 g mol^{-1}

M_w of ligands in GNRs (g mol^{-1}):

$M_w(ligand\ in\ GNRs) = N_{lig} \times M_w(ligand) = 1329816.49$ g mol^{-1}

M_w of GNR + Ligands (g mol^{-1}):

$M_w(GNRs + L) = M_w(Au\ in\ GNRs) + M_w(ligand\ in\ GNRs) = 4422088.49$ g mol^{-1}

Weight percentage of ligands in one GNR:

$wt\%\ (ligand\ in\ GNRs) = \frac{M_w(ligand\ in\ GNRs)}{M_w(GNRs)} \times 100\% = 29\%$

Supplementary Table 13 | **Calculated composition data – method B.** Dimensions, average number of gold atoms, number of ligands at the surface of the GNRs, and average weight percent of ligand shell compared to experimental TGA data.

GNR	Length / nm	Widths / nm	AR	N_{Au}	$N_{Chol\text{-}Silane}$	Wt.% ligands in GNRs	TGA / wt.%
**GNR1**	50.02	12.08	4.16	9.6×10^{3}	9.8×10^{2}	28	30
**GNR2**	50.03	19.95	2.5	1.5×10^{4}	1.7×10^{3}	29	26

Supplementary Table 14 | **Concentration (mol%) of chiral ligands and GNRs in 0.5 and 1 wt.% in 5CB.**

GNR	Length / nm	Widths / nm	Molar conc. of chiral ligands / M	Molar conc. of GNRs in 0.5 wt.% / M	Molar conc. of GNRs in 1 wt.%
**GNR1**	50.02	12.08	1.6×10^{-7}	1.9×10^{-9}	3.7×10^{-9}
**GNR2**	50.03	19.95	6.7×10^{-8}	1.3×10^{-9}	2.2×10^{-9}

Supplementary Table 15 | **Concentration (mol%) of chiral ligands and GNRs in 0.5 and 1 wt.% in 5CB.**

GNR	Length / nm	Widths / nm	M_W of GNR-chiral ligand / g mol^{-1}	Number of GNRs in LC at 0.5 wt.%	Number of GNRs in LC at 1 wt.%
**GNR1**	50.02	12.08	7.4×10^{5}	1.1×10^{15}	2.2×10^{15}
**GNR2**	50.03	19.95	1.3×10^{6}	7.8×10^{14}	2.0×10^{15}

Supplementary Table 16 | **Concentration (mol%) of chiral ligands on GNRs, helical pitch *p*, and helical twisting power β_W of the chiral ligands on the GNR surface in 5CB.**

GNR	Dimensions / nm	Wt.% of chiral ligand on GNR	*p* of N*-LC / µm at 0.5 wt.%	β_W of chiral ligands on NP / µm^{-1} wt.%$^{-1}$
**GNR1**	50.02 × 12.05	28	2.4 ± 0.79	−382.0
**GNR2**	50.03 × 19.95	29	3.4 ± 0.71	−248.0

Supplementary Table 17 | **Number of chiral ligands in N*-LC at 0.5 and 1 wt.% of GNRs in 5CB.**

**Number of chiral ligands for GNRs in 5CB per cm^3**	
**0.5 wt.%**	**1 wt.%**
**GNR1**	
3.2×10^{13}	6.6×10^{14}
**GNR2**	
2.0×10^{13}	4.1×10^{13}

Supplementary Note 5 | **Idealized *vs.* realistic ligand-shell coverage**

The siloxane-conjugation reaction starting from the in-situ prepared MPS-coated GNRs most likely leads to ligand shells that are far from the idealized version shown in Supplementary Fig. 11a. The reaction is a two-step process, hydrolysis followed by condensation catalyzed by acidic conditions (after addition of small amounts of aqueous HCl). The CH_3O—Si groups of MPS and Chol-silane **1** hydrolyze and subsequently condense to form the —Si—O—Si— network. An ideal and a perhaps more realistic model are shown below. The realistic network takes the surface curvature of the GNRs into account and shows that additional cholesterol ligands, not accounted for by the two calculation models, could lead to larger weight fractions of organic materials on the GNR surface[4]. TGA data support a model like the one shown in Supplementary Fig. 11b. Higher curvature should then lead to larger experimental values of weight loss by TGA as seen for **GNR1** in Supplementary Figure 8.

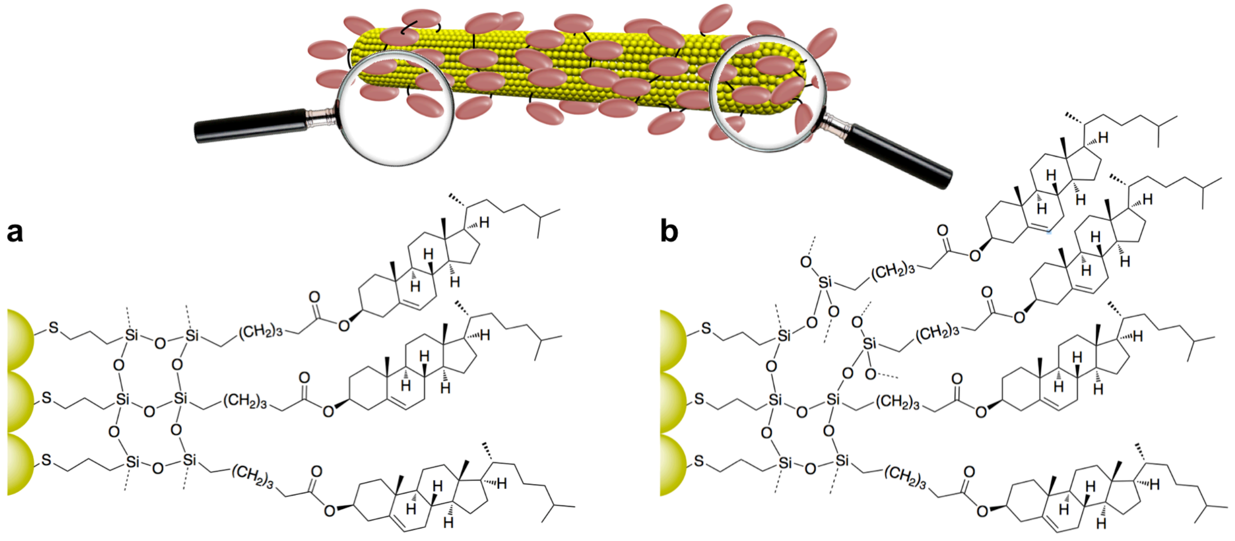

Supplementary Figure 11 | **Siloxane network. a** idealized and **b** realistic siloxane network structures considering a curved GNR surface.

Supplementary Note 6 | **Thermal analysis (DSC)**

To determine the phase transition temperatures (T in °C) and enthalpies (ΔH in J g^{-1}) of neat 5CB and 5CB doped with the GNRs we performed differential scanning calorimetry (DSC) for selected samples. The data shows that admixing the GNRs does not affect the nematic-to-isotropic ($T_{N\rightarrow Iso}$) and isotropic-to-nematic ($T_{Iso\rightarrow N}$) phase transition temperatures or enthalpies on heating or cooling, respectively. As shown in Supplementary Fig. 12, the phase transition temperatures are practically identical for the neat and doped samples.

a

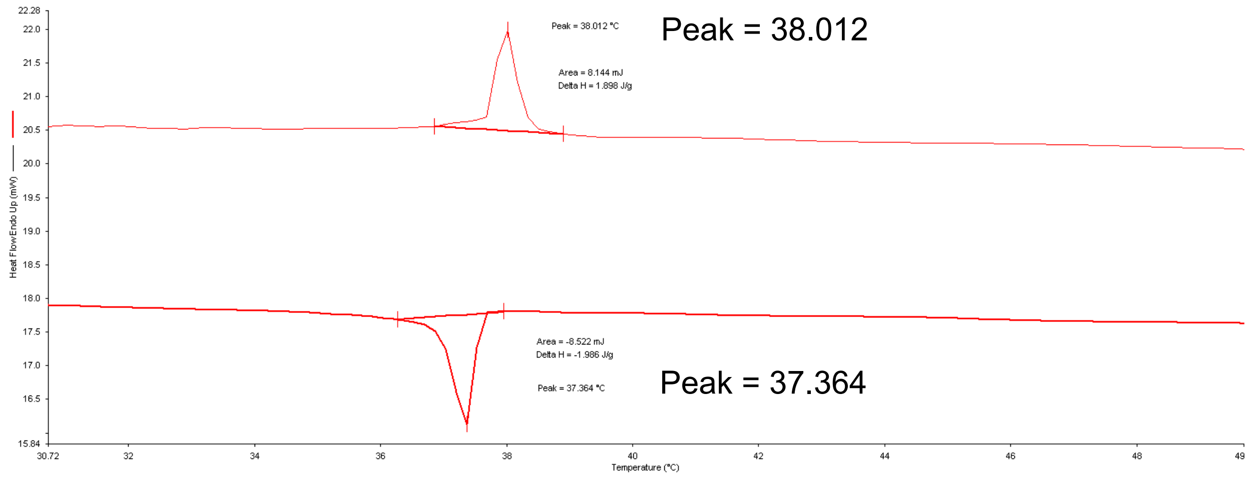

b

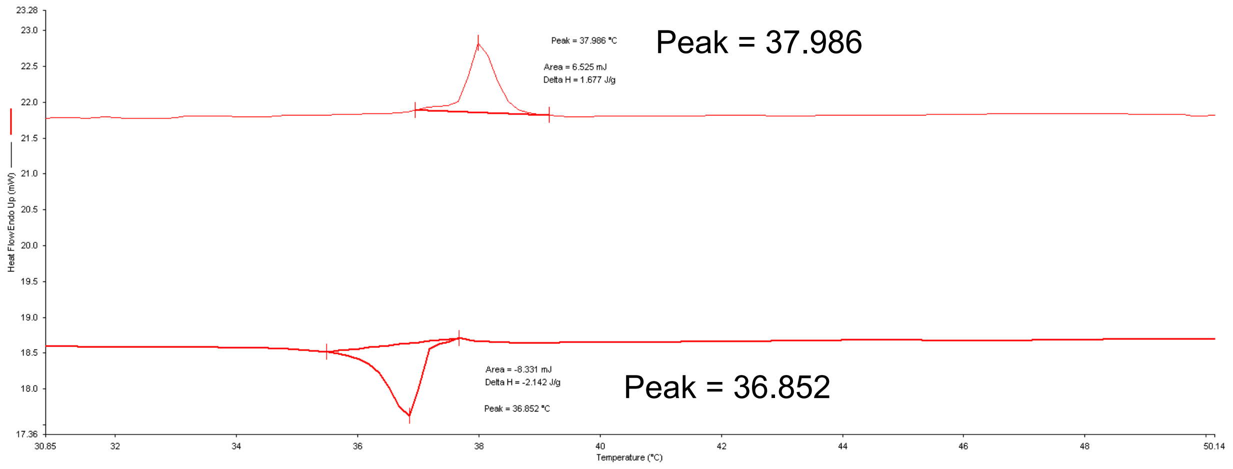

c

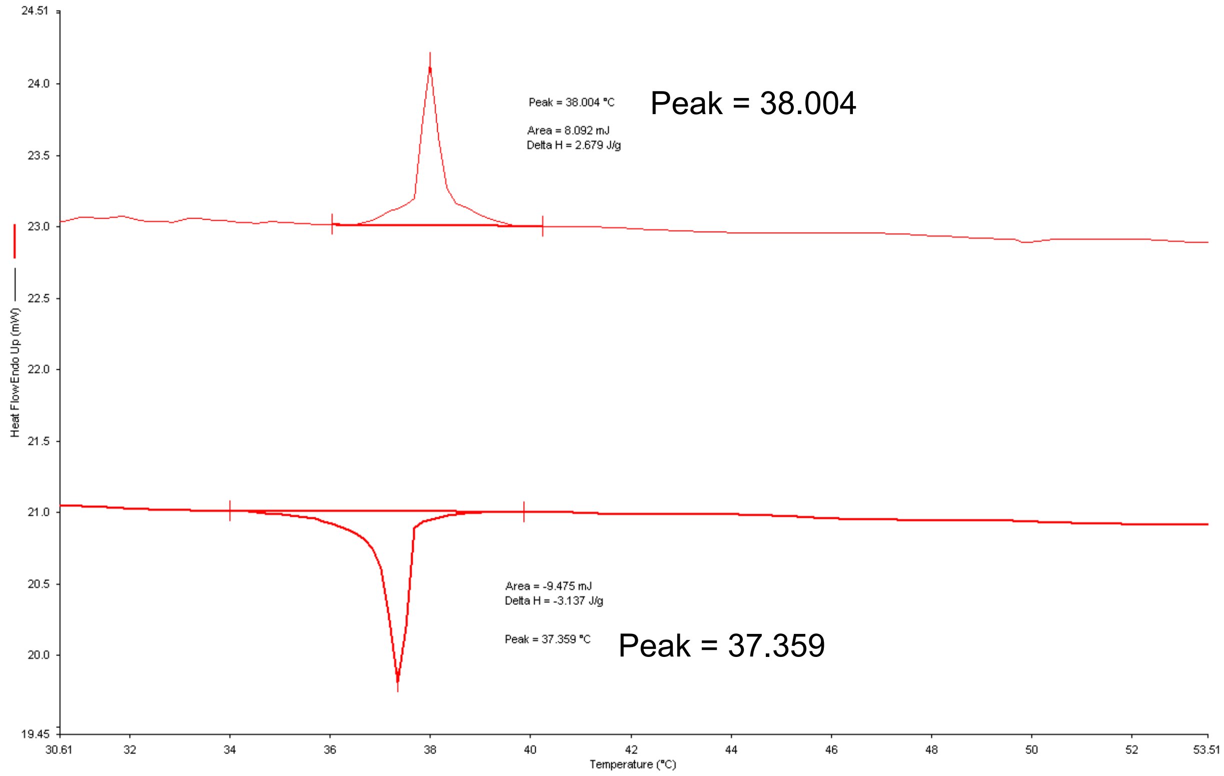

Supplementary Figure 12 | **DSC plots**. **a** Neat 5CB, **b** 5CB doped with 0.2 wt.% **GNR1**, and **c** 5CB doped with 0.5 wt.% **GNR1**. Heating/cooling rate used 10 K min^{-1}.

Supplementary Note 7 | **Handedness of induced N* LC phase**

We did not observe a discontinuation in the contact zone, and therefore the GNRs induce an N*-LC phase with a left-handed helical twist. The helical twisting power, by weight%, β_W, and the molar β_{mol} therefore carry a negative sign.

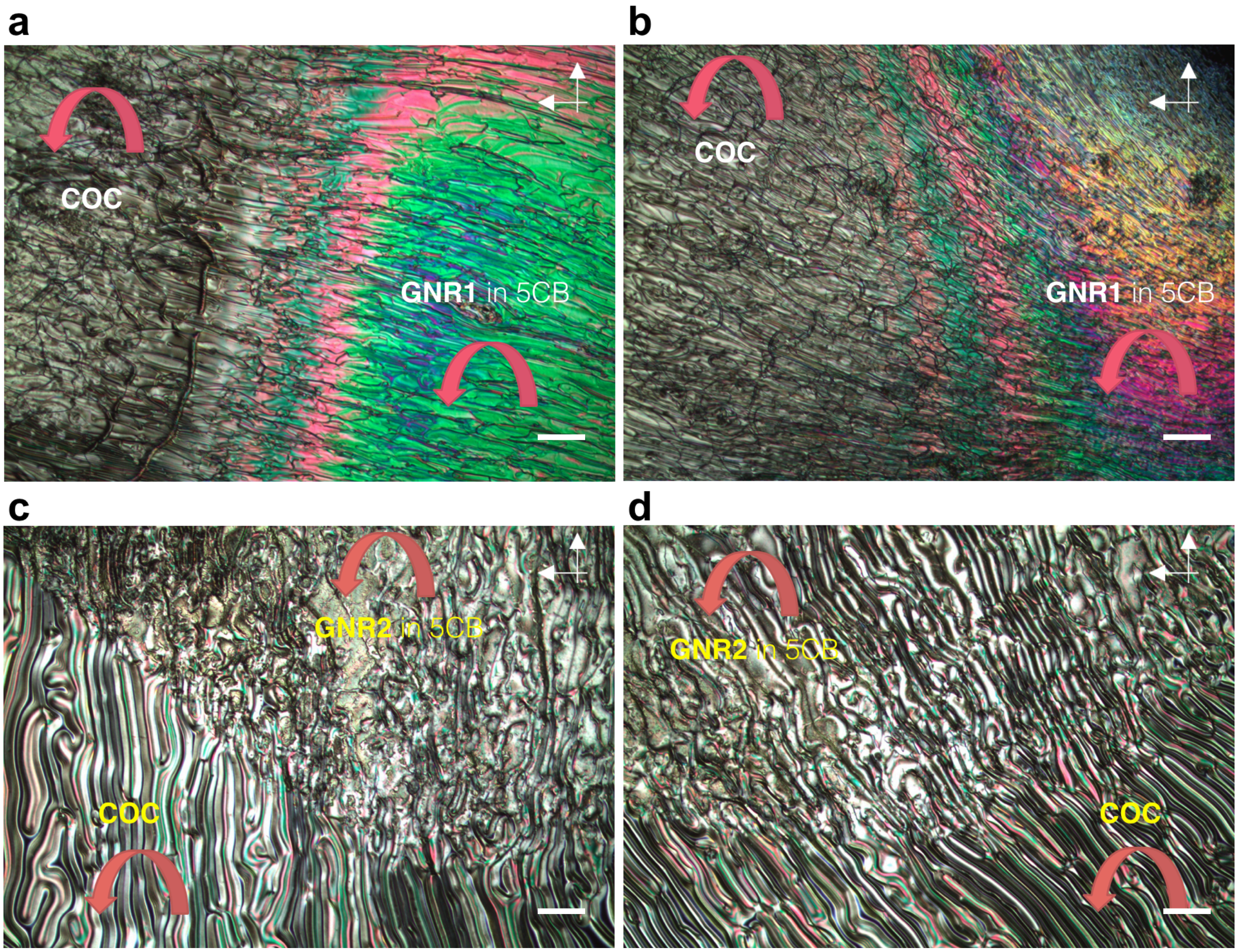

Supplementary Figure 13 | **Determination of induced N*-LC handedness.** Polarized optical photomicrographs (crossed polarizers, 20 °C) of contact preparations between: **a** and **b** COC and 5CB doped with **GNR1** (two different magnifications) and **c** and **d** COC and 5CB doped with **GNR2** (two different magnifications). Scale bars: 50 µm.

Supplementary Note 8 | **Thin film ICD spectra of N* LC films**

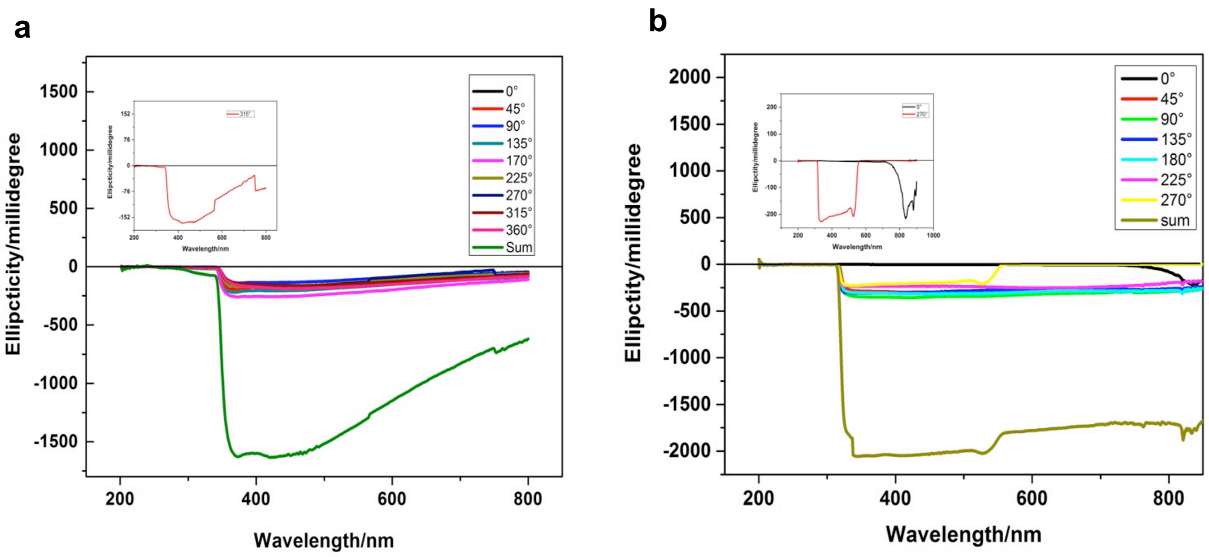

Supplementary Figure 14 | **Induced circular dichroism (ICD) spectra. a** 0.5 wt.% **GNR1** in 5CB and **b** 0.5 wt. **GNR2** in 5CB. The negative signals of the ellipticity detected for each mixture additionally confirms the induction of a left-handed N*-LC phase.

Supplementary Note 9 | **Helical pitch measurements for GNRs**

I) Free surface (bottom substrate: glass, top substrate: air)

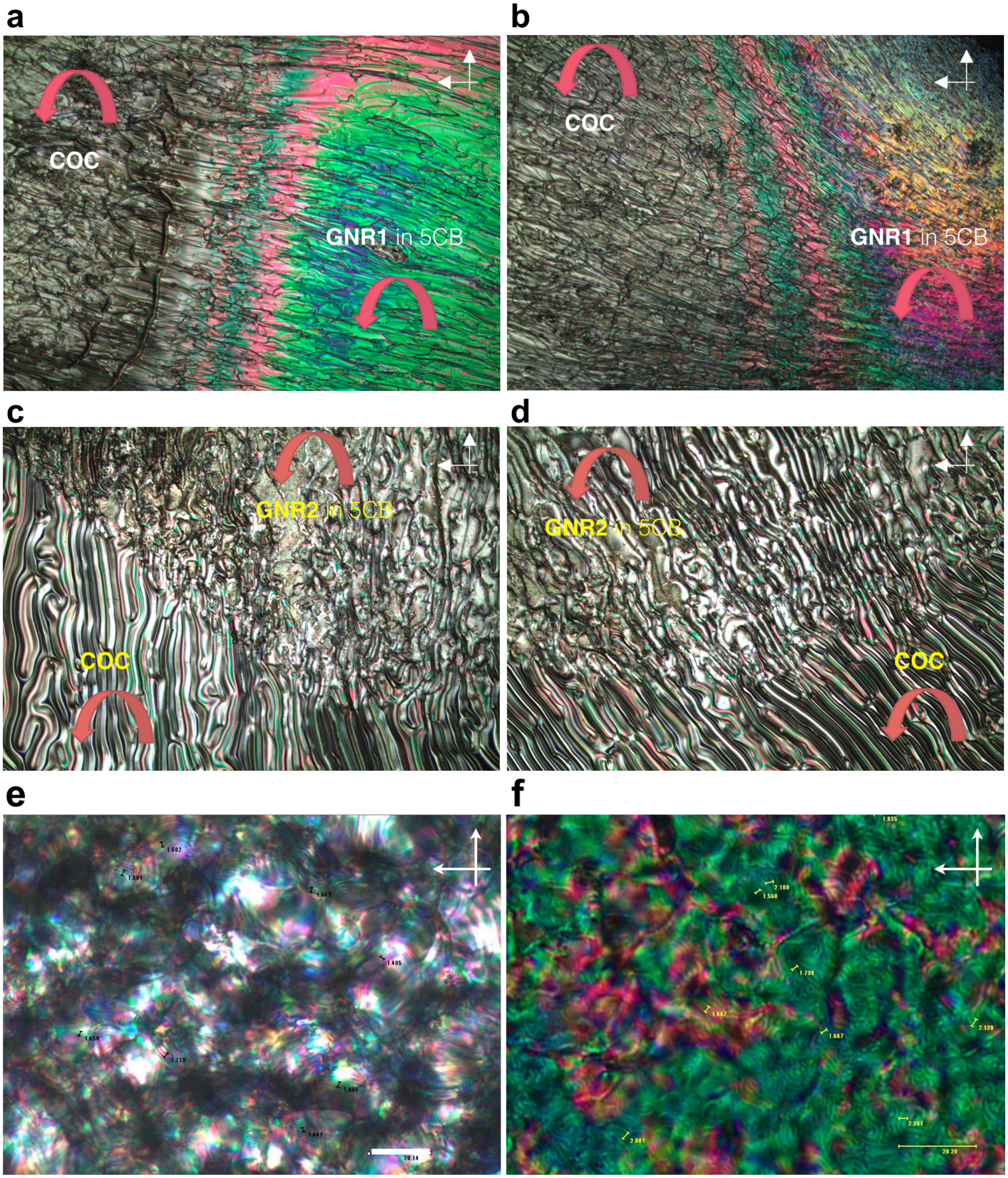

Supplementary Figure 15 | **Free-surface POM imaging to determine *p*.** Polarized optical photomicrographs (crossed polarizers, on cooling from the isotropic liquid phase) of the N*-LC

phase (bottom substrate: glass, top substrate: air) induced by **GNR1** showing fingerprint textures at the free surface with increasing concentration of **GNR1** in 5CB at 20 °C and decreasing helical pitch *p*: **a** 0.1 wt.% (scale bar: 50 μm) – inset shows a second micrograph at a different magnification (scale bar: 50 μm), **b** 0.2 wt.% (scale bar: 50 μm), **c** 0.3 wt.% (scale bar: 50 μm), **d** 0.4 wt.% (scale bar: 10 μm), **e** and **f** 0.5 wt.% (scale bar: 20 μm).

The equivalent set of images for 5CB doped with an increasing concentration of **GNR2** is shown in Fig. 3h – 3l. Supplementary Fig. 16 and Supplementary Fig. 17 show the plots of the inverse helical pitch ($1/p$ in μm^{-1}) measured using these free surface preparations *vs.* the concentration (wt.%) of the dispersed GNRs.

a

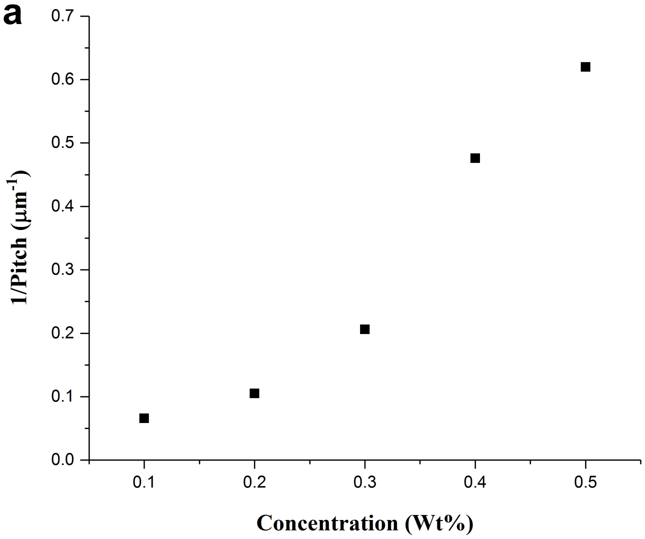

b

Concentration / wt.%	1/*p* / μm^{-1}
0.1	0.066
0.2	0.105
0.3	0.206
0.4	0.476
0.5	0.62

Supplementary Figure 16 | **Calculation of β_W from free surface images. a** Plot of inverse pitch $1/p$ (μm^{-1}) of induced N*-LC phase at free surface induced by doping 5CB with different concentrations of **GNR1** *vs.* concentration of the GNRs (wt.%). The plot shows a slight deviation from a linear relationship, where the inverse helical pitch consistently increases with increasing concentration of the GNRs. **b** Data for plot from images shown in Supplementary Fig. 15.

a

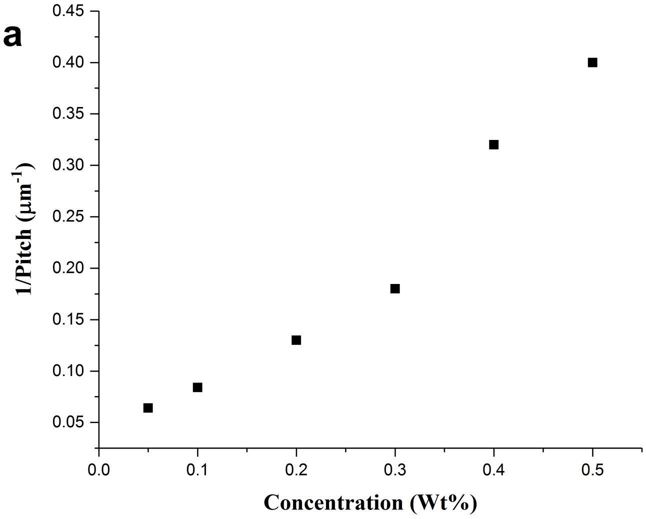

b

Concentration / wt.%	1/*p* / μm^{-1}
0.05	0.064
0.1	0.084
0.2	0.129
0.3	0.208
0.4	0.328
0.5	0.40

Supplementary Figure 17 | **Calculation of β_W from free surface images. a** Plot of inverse pitch $1/p$ (μm^{-1}) of induced N*-LC phase at free surface induced by doping 5CB with different concentrations of **GNR2** *vs.* concentration of the GNRs (wt.%). The plot shows an almost linear relationship, where the inverse helical pitch consistently increases with increasing concentration of the GNRs. **b** Data for plot from images in Figs. 3h – 3l.

II) Grandjean-Cano wedge cells

a

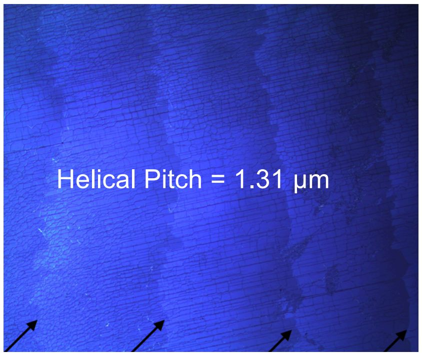

b

**# of Cano lines**	***Distance* / μm**
1	4.56
2	5.34
3	5.78
4	6.55
5	7.21
6	7.88

Number of Cano's lines

c

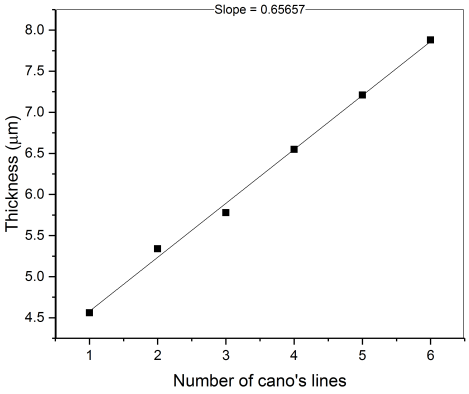

Supplementary Figure 18 | **Determination of *p* using Cano wedge cell. a** POM image of Grandjean Cano wedge cell (crossed polarizers) showing Grandjean steps (lines) of the induced N*-LC phase after doping with 0.7 wt.% **GNR1**, **b** data from POM image analysis, and **c** plot of distance between Cano lines (thickness) *vs*. number of lines.

a

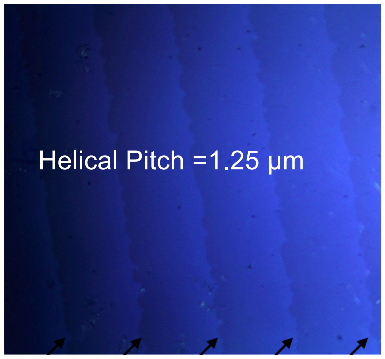

b

**# of Cano lines**	***Distance* / μm**
1	7.69
2	8.29
3	8.80
4	9.68
5	10.18
6	10.77

c

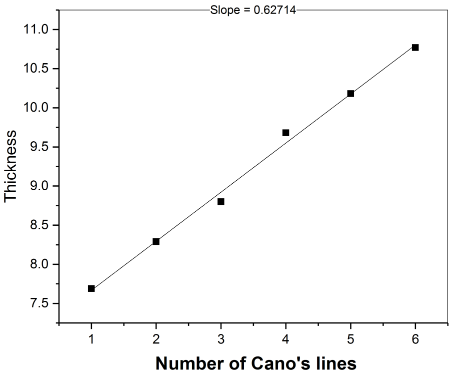

Supplementary Figure 19 | **Determination of *p* using Cano wedge cell. a** POM image of Grandjean Cano wedge cell (crossed polarizers) showing Grandjean steps (lines) of the induced N*-LC phase after doping with 0.6 wt.% **GNR1**, **b** data from POM image analysis, and **c** plot of distance between Cano lines (thickness) *vs*. number of lines.

a

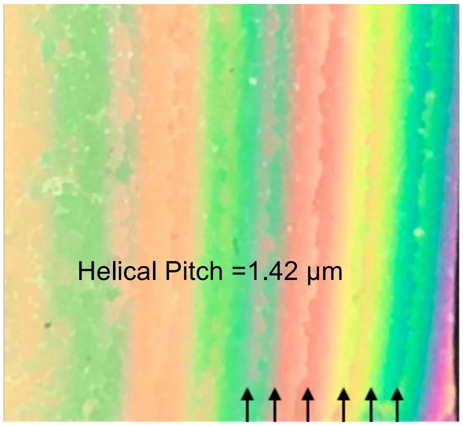

b

# of Cano lines	*Distance* / µm
1	16.37
2	17.21
3	17.84
4	18.47
5	19.30

c

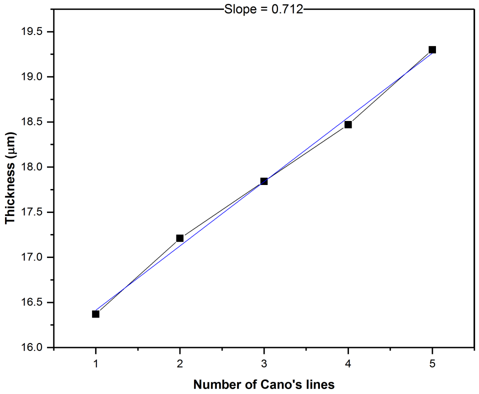

Supplementary Figure 20 | **Determination of *p* using Cano wedge cell. a** POM image of Grandjean Cano wedge cell (crossed polarizers) showing Grandjean steps (lines) of the induced N*-LC phase after doping with 0.5 wt.% **GNR1**, **b** data from POM image analysis, and **c** plot of distance between Cano lines (thickness) *vs*. number of lines.

a

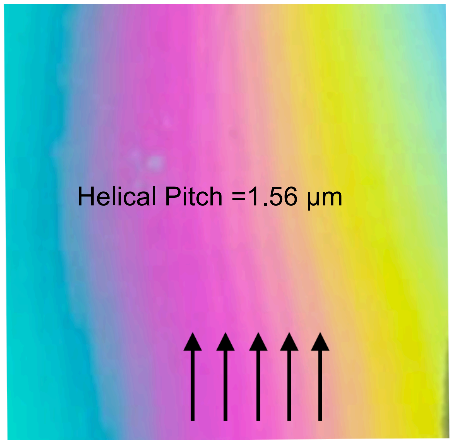

b

# of Cano lines	*Distance* / µm
1	0.33
2	1.15
3	2.15
4	2.64
5	3.47
6	4.3

c

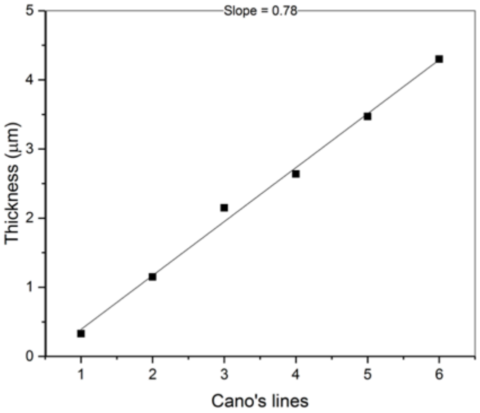

Supplementary Figure 21 | **Determination of *p* using Cano wedge cell. a** POM image of Grandjean Cano wedge cell (crossed polarizers) showing Grandjean steps (lines) of the induced N*-LC phase after doping with 0.4 wt.% **GNR1**, **b** data from POM image analysis, and **c** plot of distance between Cano lines (thickness) *vs*. number of lines.

a

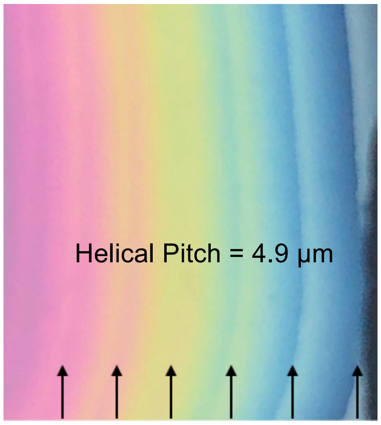

b

# of Cano lines	*Distance /* μm
1	2.90
2	5.70
3	8.40
4	11.30
5	13.40
6	16.0
7	18.25
8	20.20

c

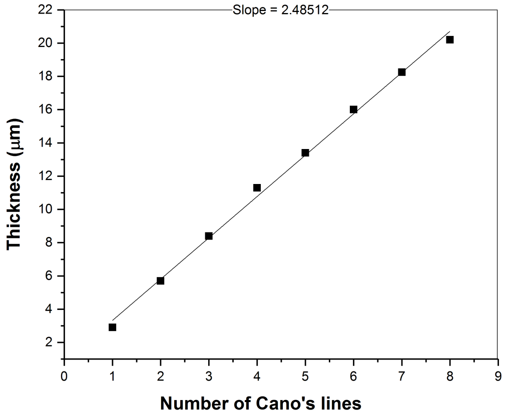

Supplementary Figure 22 | **Determination of *p* using Cano wedge cell. a** POM image of Grandjean Cano wedge cell (crossed polarizers) showing Grandjean steps (lines) of the induced N*-LC phase after doping with 0.35 wt.% **GNR1**, **b** data from POM image analysis, and **c** plot of distance between Cano lines (thickness) *vs*. number of lines.

a

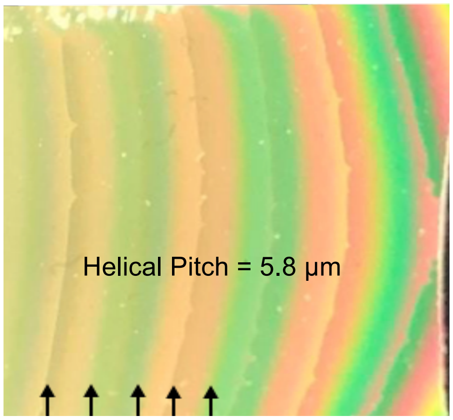

b

# of Cano lines	*Distance /* μm
1	6.43
2	8.56
3	11.49
4	14.82
5	18.02

c

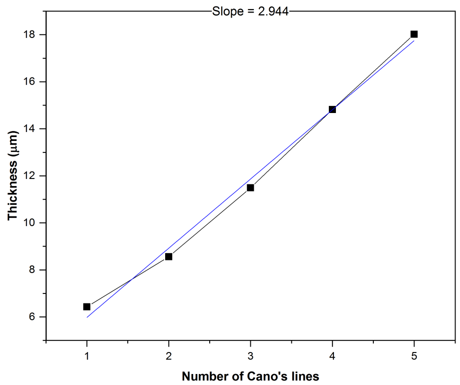

Supplementary Figure 23 | **Determination of *p* using Cano wedge cell. a** POM image of Grandjean Cano wedge cell (crossed polarizers) showing Grandjean steps (lines) of the induced N*-LC phase after doping with 0.3 wt.% **GNR1**, **b** data from POM image analysis, and **c** plot of distance between Cano lines (thickness) *vs*. number of lines.

a **b** **c**

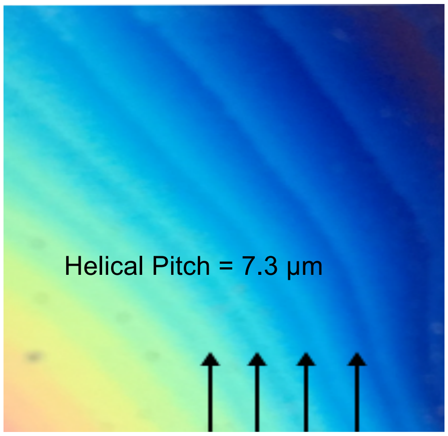

# of Cano lines	*Distance* / µm
1	10.47
2	14.50
3	18.20
4	22.40
5	25.45
6	28.34

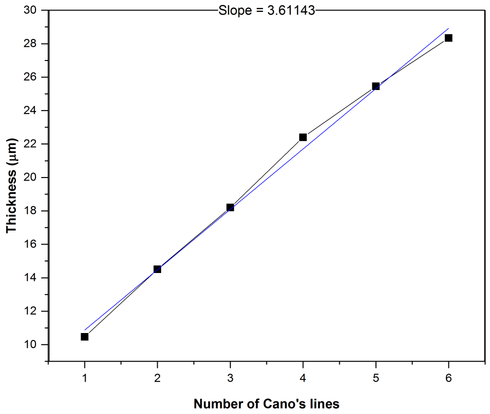

Supplementary Figure 24 | **Determination of *p* using Cano wedge cell. a** POM image of Grandjean Cano wedge cell (crossed polarizers) showing Grandjean steps (lines) of the induced N*-LC after doping with 0.2 wt.% **GNR1**, **b** data from POM image analysis, and **c** plot of distance between Cano lines (thickness) *vs*. number of lines.

a **b** **c**

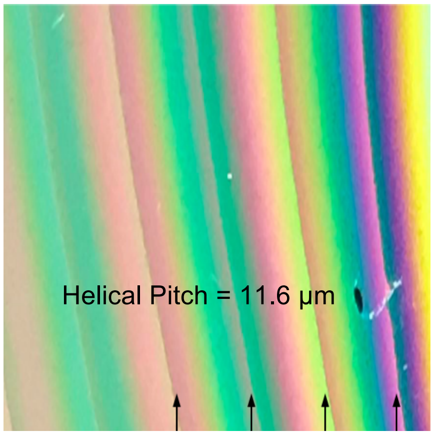

# of Cano lines	*Distance* / µm
1	13.81
2	18.39
3	23.42
4	31.26
5	36.85

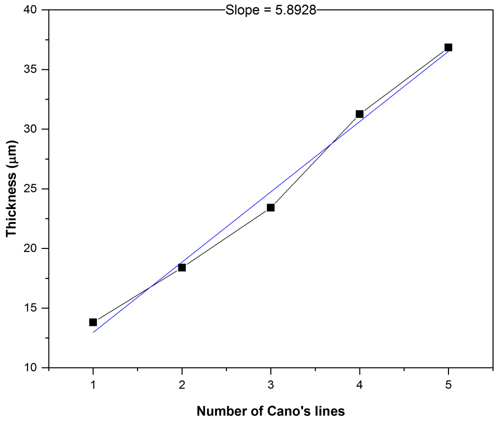

Supplementary Figure 25 | **Determination of *p* using Cano wedge cell. a** POM image of Grandjean Cano wedge cell (crossed polarizers) showing Grandjean steps (lines) of the induced N*-LC phase after doping with 0.1 wt.% **GNR1**, **b** data from POM image analysis, and **c** plot of distance between Cano lines (thickness) *vs*. number of lines.

a

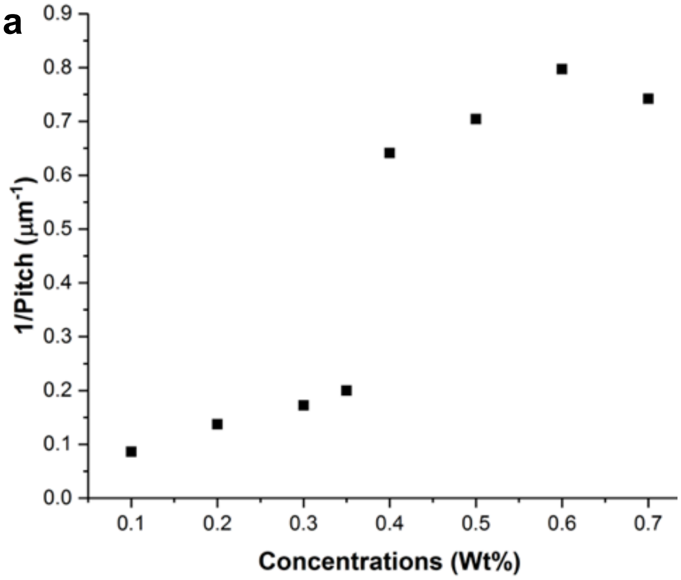

b

Concentration / wt.%	*1/p* / μm^{-1}
0.1	0.086
0.2	0.137
0.3	0.172
0.35	0.200
0.4	0.641
0.5	0.704
0.6	0.800
0.7	0.763

Supplementary Figure 26 | **Calculation of β_W from Cano wedge cell measurements. a** Plot of inverse helical pitch $1/p$ (μm^{-1}) *vs.* concentration of **GNR1** in 5CB (wt.%). The plot shows a deviation from the expected linear relationship of inverse pitch *vs*. concentration for the highest concentration indicating that at this higher concentration of **GNR1** in 5CB (at 0.5 wt.%) either the helical pitch of the induced N*-LC phase saturates, or that the GNRs start to aggregate at this higher concentration, or that both scenarios described are active. **b** Data for plot (images in Figs. 3m – 3q and Supplementary Figs. 18 – 25).

Comparing the plots of $1/p$ *vs.* wt.% **GNR1** in 5CB (Supplementary Figs. 16 and 26) validates that the two methods of measuring the helical pitch (free surface and Cano wedge method) in our experiments produced very similar results, and that each dataset can be used to calculate the helical twisting power. The plot in Supplementary Fig. 26 also shows at a concentration of 0.7 wt.% of **GNR1** in 5CB an onset of aggregation of the GNRs, indicated by the sudden drop of $1/p$.

Supplementary Note 10 | **Helical pitch measurements: COC and ZLI-4572 in 5CB**

a

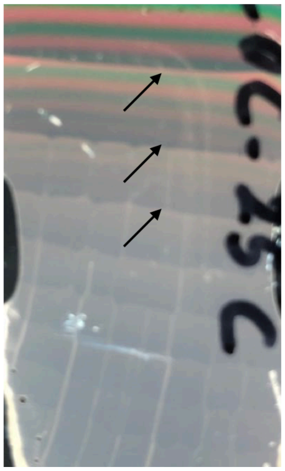

b

Cano's Lines	Thickness
1	18.58
2	29.14
3	37.64
4	44.08
5	50
6	55.16
7	60.05

c

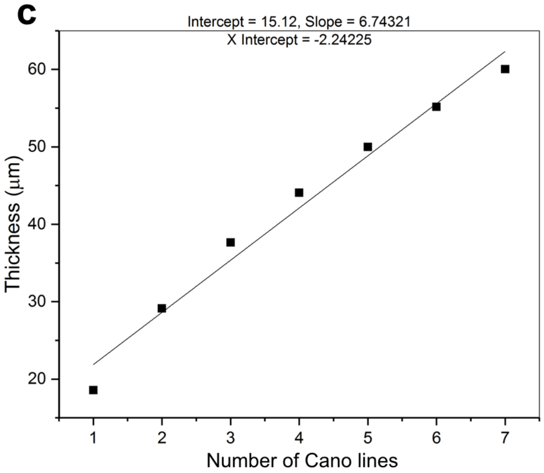

Supplementary Figure 27 | **Determination of *p* using Cano wedge cell. a** POM image of Grandjean Cano wedge cell (crossed polarizers) showing Grandjean steps (lines) of the induced N*-LC phase after doping with 1.0 wt.% **COC**, **b** data from POM image analysis, and **c** plot of distance between Cano lines (thickness) *vs*. number of lines.

a

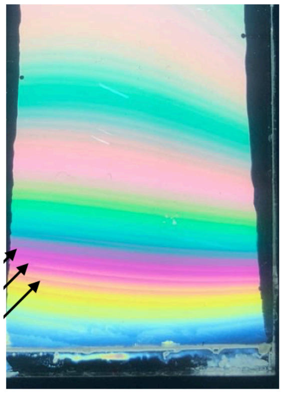

b

Cano's Lines	Thickness
1	6.4
2	8.39
3	10.38
4	11.7
5	13

c

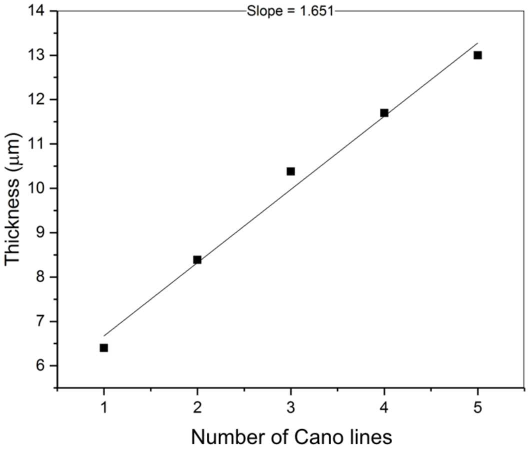

Supplementary Figure 28 | **Determination of *p* using Cano wedge cell. a** POM image of Grandjean Cano wedge cell (crossed polarizers) showing Grandjean steps (lines) of the induced N*-LC phase after doping with 1.0 wt.% **ZLI-4572**, **b** data from POM image analysis, and **c** plot of distance between Cano lines (thickness) *vs*. number of lines.

Supplementary Note 11 | **Persistence length calculation (inter-particle distance)**

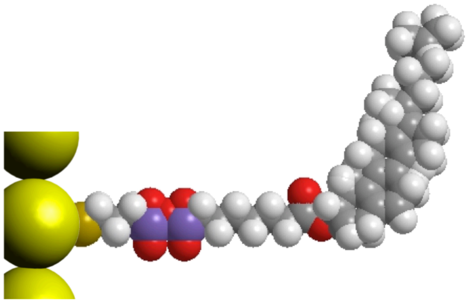

Supplementary Figure 29 | **Space filling model.** Chol-silane **1** conjugated to MPS-capped GNRs in the conformation used for calculations.

Si_{Chol}: Cross-section of Chol-Si = 0.7 nm^2

S_{thiol}: Surface area of thiol on gold surface = 0.21 nm^2

Length of Chol-Si = 2.56 nm

Density Chol-Si: 1.067 g/cm^3

Density 5CB: 1.008 g/cm^3

D_{GNRs}: Diameter of the GNRs = D + 2L Chol-Si

$$Number\ of\ 5CB\ around\ GNRs\ = \frac{1}{mol\%\ of\ GNRs\ in\ 5CB}$$

$$Distance\ of\ GNRs = \sqrt[3]{\frac{2\times\ Mw\ of\ 5CB \times Number\ of\ 5CB\ around\ GNRs}{Avagadro\ Number \times \rho\ 5\mathrm{CB}}} - D_{GNRs}$$

$$Number\ of\ 5CB\ around\ GNRs = 2{\times}10^5$$

$$D_{\boldsymbol{GNR1}} = 10 + 2{\times}2.5 = 15\ nm$$

$$Distance\ of\ \boldsymbol{GNR1} = 54 - 15 = \boldsymbol{39\ nm}$$

$$Number\ of\ 5CB\ around\ GNRs = 1{\times}10^6$$

$$D_{\boldsymbol{GNR2}} = 23 + 2{\times}2.5 = 27nm$$

$$Distance\ of\ \boldsymbol{GNR2} = 93 - 27 = \boldsymbol{66\ nm}$$

Supplementary Note 12 | **Felix-2900-03 – a different N-LC host**

To test that the p values are not unique to 5CB, we also tested another N-LC host, Felix-2900-03. As shown in Supplementary Fig. 24, almost identical p values were found at the same reduced temperature ($T_{Iso \to N} - T$ = 15 °C) on cooling.

a **b**

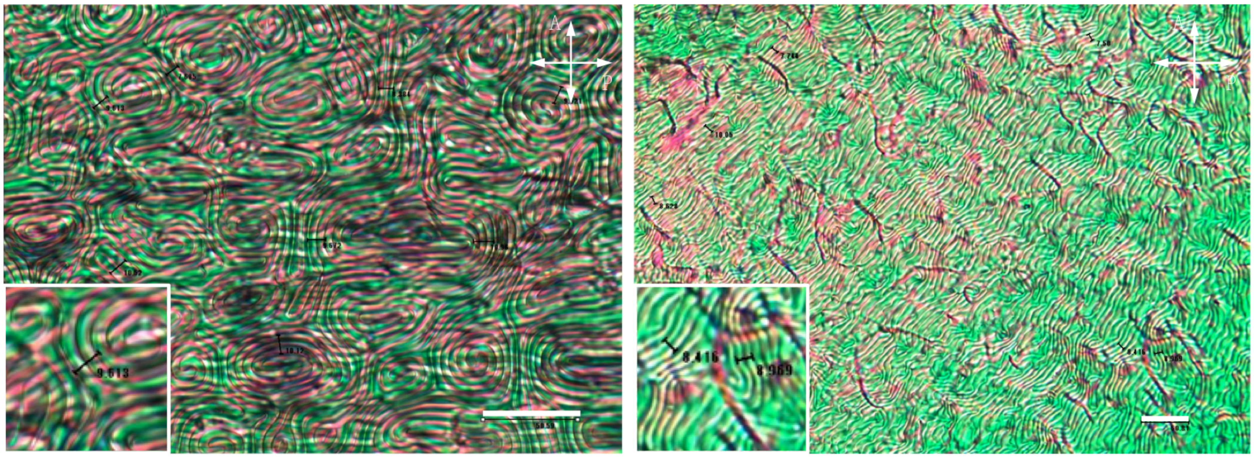

c

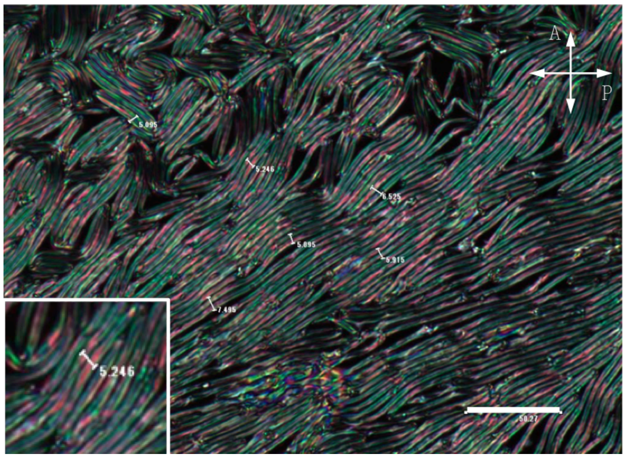

d

wt.% **GNR1**	p / µm
0.1	9.5
0.2	8.3
0.3	5.9

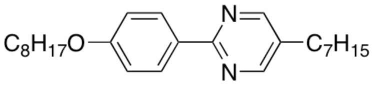

FELIX-2900-03; Cr 52 (SmA 45) **N** 70 Iso [°C]

Supplementary Figure 30 | **POM micrographs of induced N*-LC phase of Felix-2900-03 doped with GNR1: a** 0.1 wt.%, **b** 0.2 wt.%, and **c** 0.3 wt.%. Scale bar in each image: 50 µm, crossed polarizers, taken at $T_{Iso \to N} - T$ = 15 °C. Insets show magnified areas of each image with overlaid microruler. **d** Summary of p (µm) *vs*. concentration (wt.%) data and chemical structure as well as phase sequence and transition temperatures of Felix-2900-03.

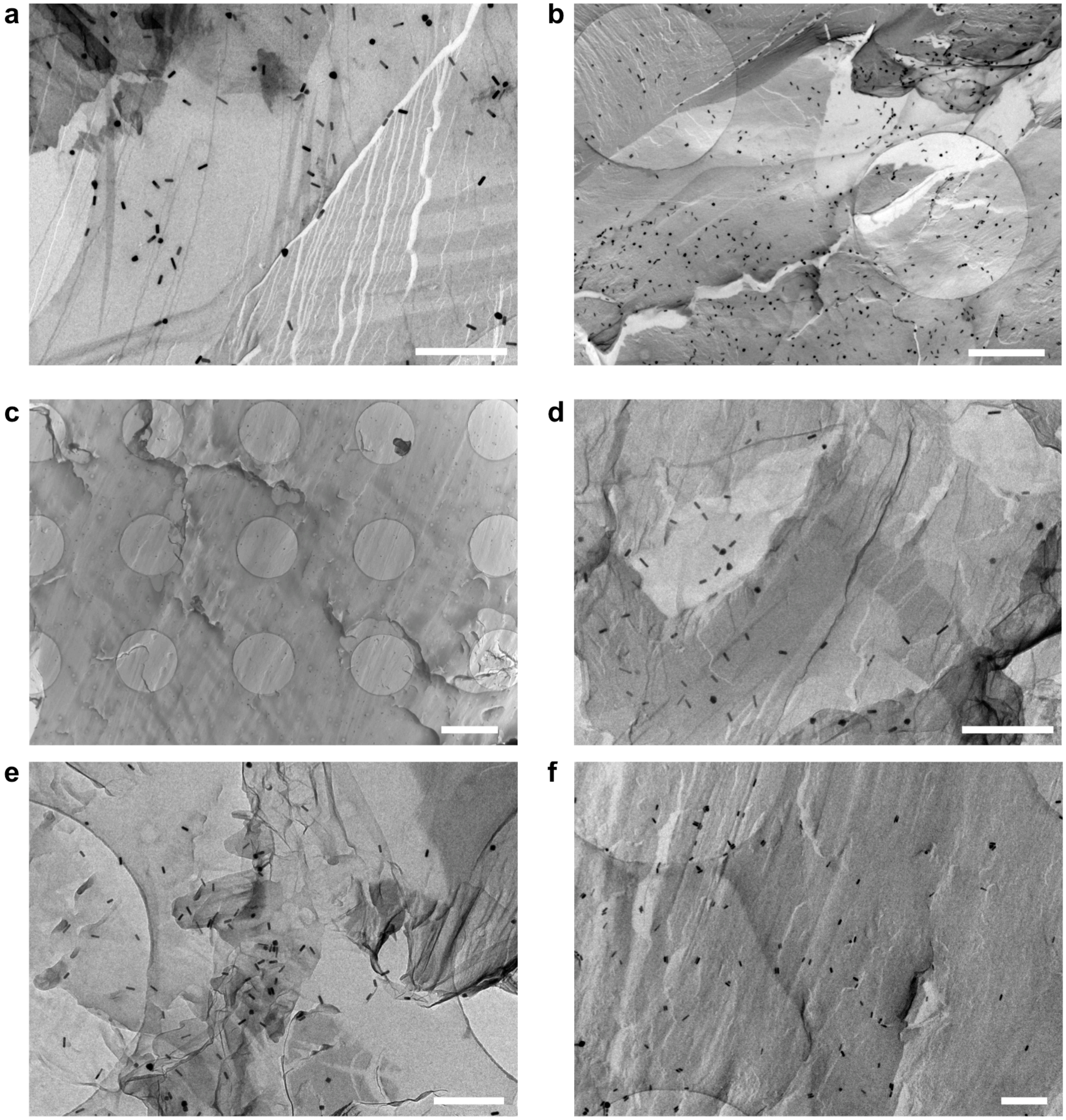
a
b
c
d
e
f

g h

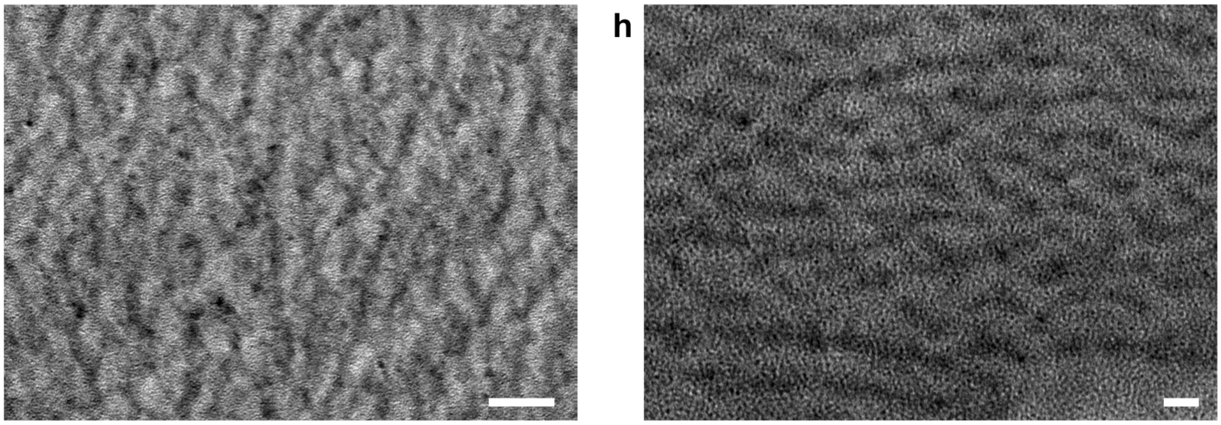

Supplementary Figure 31 | **FF-TEM images of multi-domain N*-LC droplets on glass: a – f** Various sections of different grids and sample preparations of 0.5 wt.% **GNR2** in Felix-2900-03 (the circles in different grey scale are from the TEM grids; scale bars: **a** 500 nm, **b** 1 μm, **c** 2 μm, **d – f** 500 nm). Although some images show occasional side-by-side and end-to-end stacking of pairs of GNRs, the out-zoomed images shown in **b** and **c** support the argument that the GNRs do not significantly aggregate in the induced N*-LC. **g** and **h** FF-TEM images of mixtures of 0.5 wt.% **NP2** (∅ = 5.5 nm) in Felix-2900-03, prepared exactly as the GNR in Felix-2900-03 samples (scale bars: 20 nm). Again, the separation of the well-dispersed NPs matches in several areas of these FF-TEM samples the calculated $D_{\mathrm{P\text{-}P}}$ values listed in Table 1 and plotted in Fig. 7e.

Supplementary Note 14 | **Capped particles chirality quantification**

Details of the calculation of the chirality indicator G_{oa}^{a} are given in Eq.1 of the main text[8]. We have first build atomistically detailed models of both GNRs and the quasi-spherical Au NPs as well as of the Chol-silane ligands. As expected all GNRs and NPs turn out to be non chiral ($G_{oa}^{a} = 0$), while the ligand molecule is characterized by a value of $G_{oa}^{a} = -2004.7$.

In the experiments, the ligands are firmly attached to the GNR or Au NP surface, but still their conformation and orientation can change. Moreover, the experiment involves a certain number of GNRs and NPs. We thus performed the calculation of G_{oa}^{a} for a number of likely possibilities sufficiently large to be statistically significant and then took an average. In order to keep this manageable, particularly in terms of computer time, a number of simplifications have been considered. In fact, due to the very large number of atoms involved, these computations for the decorated GNRs and NPs are so highly demanding to suggest moving from an atomistic description to a coarse-grained (CG) one. In this CG representation we have retained only the positions of 5 atoms of the TCS, taken from the free molecule conformation of minimum energy obtained with the Universal Force Field[9] to provide a minimal description of the ligand structure, as illustrated in main text Figs. 8a and 8b. The overall chirality index value of the Chol-silane ligand at a CG level is $G_{oa}^{a} = -0.295$, with the same sign of the atomistic ligand chirality value, even if the absolute value is 4 order of magnitude smaller. In practice each CG ligand molecule has been assigned a certain position and orientation with respect to the NP coordinate frame of both spherocylindrical and spherical nanoparticles. As for the NPs, being achiral themselves, only the proper enveloping shape determining the ligand positions and the center of mass have been considered. The orientations, positions, and the number of Chol-silane ligands bound (1, 2, 3, 5, 10, and 20) have then been varied. In Figs. 8c and 8d we show an example of a GNR and a Au NP with 20 ligand molecules randomly bound.

Supplementary Figure 32 describes the trends of the absolute value of average chirality index ($| < G_{oa}^{a} > |$), obtained by considering 20k configurations for each system, where the position and the orientation have been chosen randomly, only avoiding superpositions of the CG ligands. We see that increasing the number of chiral ligands bound to the GNRs and NPs generally increases the overall chirality index, but the main finding is that the ligand decorated **GNRs** correspond to a higher chirality with respect to that of the Au NPs and that the aspect ratio of the nanorod is directly proportional to the G_{oa}^{a}, as found experimentally (see Table 1). We estimate that 20k random configurations should provide an adequate sampling for the relatively

small Au NPs. However, since the number of non-overlapping ligand configurations for the nanorods to be capped with 20 ligands of the dimensions given is much higher, we have also followed another procedure, selecting a limited number of positions and orientations and computing the G_{oa}^{a} for all the possible resulting combinations. The positions chosen are: one at the top and one at the bottom end of the GNRs and 20 in the middle of the rod. Only the angle α between the ligand's main axis and the long axis of the nanorod is allowed to vary (values $\alpha = 0°$ or $\alpha = 60°$), assigning the same value of α to the same group of ligands. This selection of tilt angles and positions leads to a manageable number of 1848 possible combinations for the GNRs that are taken into account to produce the results in Fig. 9 of the main text.

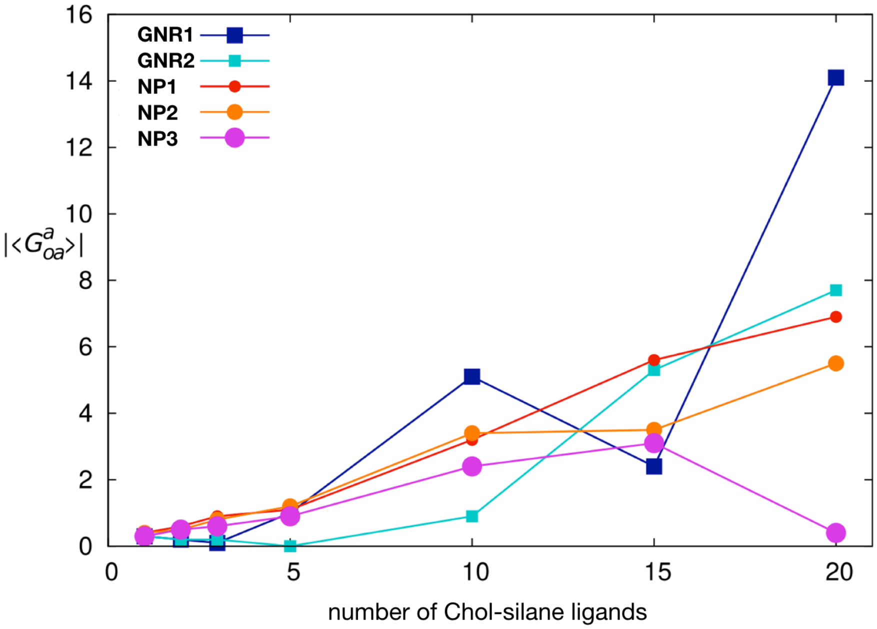

Supplementary Figure 32 | **Trends of | $< G_{oa}^{a} >$ | with respect to the number of CG Chol-silane ligands bound to the GNRs and Au NPs.** Absolute average overall chirality (| $< G_{oa}^{a} >$ |) for 20k configurations of NPs capped with an increasing number of Chol-silane ligand molecules.

References

1. Sharma, A., Mori, T., Lee, H.-C., Worden, M., Bidwell, E., Hegmann, T. Detecting, visualizing, and measuring gold nanoparticle chirality using helical pitch measurements in nematic liquid crystal phases. *ACS Nano* **8**(12), 11966-11976 (2014).

2. Nikoobakht, B., El-Sayed, M. A. Preparation and growth mechanism of gold nanorods (NRs) using seed-mediated growth method. *Chem. Mater.* **15**(10), 1957-1962 (2003).

3. Umadevi, S., Feng, X., Hegmann, T. Large area self-assembly of nematic liquid-crystal-functionalized gold nanorods. *Adv. Funct. Mater.* **23**(11), 1393-1403 (2013).

4. Mirzaei, J., Urbanski, M., Kitzerow, H.-S., Hegmann, T. Synthesis of liquid crystal silane-functionalized gold nanoparticles and their effects on the optical and electrooptic properties of a structurally related nematic liquid crystal. *ChemPhysChem* **15**(7), 1381-1394 (2014).

5. Feng, X., Sosa-Vargas, L., Umadevi, S., Mori, T., Shimizu, Y., Hegmann, T. Discotic liquid crystal-functionalized gold nanorods: 2- and 3D self-assembly and macroscopic alignment as well as increased charge carrier mobility in hexagonal columnar liquid crystal hosts affected by molecular packing and pi-pi interactions. *Adv. Funct. Mater.* **25**(8), 1180-1192 (2015).

6. Mori, T., Sharma, A., Hegmann, T. Significant enhancement of the chiral correlation length in nematic liquid crystals by gold nanoparticle surfaces featuring axially chiral binaphthyl ligands. *ACS Nano* **10**(1), 1552-1564 (2016).

7. Schneider, C. A., Rasband, W. S., Eliceiri, K. W. NIH Image to ImageJ: 25 years of image analysis. *Nat. Methods* **9**(7), 671-675 (2012).

8. Pietropaolo, A., Muccioli, L., Berardi, R., Zannoni, C. A chirality index for investigating protein secondary structures and their time evolution. *Proteins* **70**, 667-677 (2008).

9. Rappe, A. K., Casewit, C. J., Colwell, K. S., Goddard, W. A., Skiff, W. M. UFF, a full periodic table force field for molecular mechanics and molecular dynamics simulations. *J. Amer. Chem. Soc.* **114**, 10024-10035 (1992).

```
clear all
close all
warning('off')
% some Paramters for quick look at intensity histogram.
threshold=140;   % cutoff value to select background and Object.
numOfPx =100;    % Remove objects containing fewer than 'numOfPx' pixels.
numOfPx can change.
Y = [20 20]; % scale position
X=  [20 70];
%-----------------------------------------------------------
% Load the data and assign some variables
workingDir         =pwd; % where the program lies.
[fileName,pathName]=uigetfile('*.JPG','Select ".JPG" file');
cd(pathName);
% If we get here, we should have found the image file.
ImgDATA            = imread(fileName);
cd(workingDir)
%channel            =input('which channel do you want to use? 1(=red),
2(=green) or 3(=blue) ');
%I                  =ImgDATA(:,:,channel);

gray= rgb2gray(ImgDATA);
%figure, imshow(gray)
K = medfilt2(gray);
imshowpair(gray,K,'montage')
change = adapthisteq(K,'clipLimit',0.02,'Distribution','rayleigh');
I2 = wiener2(K,[18 18]);

%-----------------------------------------------------------
% plot the original Image
figure(1);
%imshow(ImgDATA)
imshowpair(ImgDATA,I2,'montage')
fontsize=14;
title('Original Image','FontSize',fontsize)
line(X,Y,'Color','r','LineWidth',3)
%-----------------------------------------------------------
% Do some processing
% Adjust the image. Use imadjust to increase the contrast of the processed
image I2 by saturating 1% of
% the data at both low and high intensities and by stretching the intensity
values to fill the
% uint8 dynamic range.
%I2=imadjust(I);
%-----------------------------------------------------------
% Create a binary image of the processed image
binaryImage = I2 < threshold;
bw3 = bwareaopen(binaryImage, numOfPx); % very important
bw4 = imfill(bw3, 'holes');
%-----------------------------------------------------------
% plot the processed image
figure(2)
```

```
imshow(bw4)% Do a "hole fill" to get rid of any background pixels or "holes"
inside the connected region.
title('Binary Image, obtained by thresholding, and removing small connected
objects and filling');
%axis image; % Make sure image is not artificially stretched because of
screen's aspect ratio.
hold on;
% create the boundary of objects and plot on the same figure
boundaries = bwboundaries(bw4);
numberOfBoundaries = size(boundaries, 1);

for k = 1 : numberOfBoundaries
    thisBoundary = boundaries{k};
    plot(thisBoundary(:,2), thisBoundary(:,1),'g', 'LineWidth', 2);
end

line(X,Y,'Color','r','LineWidth',3)
hold off;
%------------------------------------------------------------
% Do some anlysis
% Find all the connected components (objects) in the binary image. 4 is
% connectivity. It can assume 4, 8 or arbitrary. The accuracy of your results
depends on the size of the objects, the connectivity parameter (4, 8, or
arbitrary),
% and whether or not any objects are touching (in which case they could be
labeled as one
% object). Some of the rice grains in the binary image bw are touching.

cc = bwconncomp(bw4, 4); % cc=connected component, 8:connected neighborhood
% Connectivity defines which pixels are connected to other pixels. A set of
pixels in a binary image that form a connected group is called an object or a
connected component.
numberOfObjects=cc.NumObjects;
fprintf('Number of objects= %5d\n',numberOfObjects);
%------------------------------------------------------------
labeled = labelmatrix(cc);
RGB_label = label2rgb(labeled, 'hsv', 'k', 'shuffle');
figure(3)
imshow(RGB_label)
title('Pseudocolor image of objects','FontSize',fontsize)

line(X,Y,'Color','r','LineWidth',3)
%------------------------------------------------------------
spot_properties  = regionprops(cc, 'all');
numberOfSpots    = size(spot_properties,1);
%------------------------------------------------------------
blobECD = zeros(1, numberOfSpots); % ECD=equivalent circular diameter
meanGL          =zeros(numberOfSpots,1);
spot_areas      =[spot_properties.Area]';
 fprintf(1,'Blob #      MajorAxisLength  MinorAxisLength   AspectRatio\n');
 labelShiftX=0;
for k = 1 : numberOfSpots
    spot_centroid              =spot_properties(k).Centroid;
    spot_MajorAxisLength       = spot_properties(k).MajorAxisLength;
% Get MajorAxisLength
```

```
    spot_MinorAxisLength       = spot_properties(k).MinorAxisLength;
% Get MinorAxisLength
    Aspect_ratio =             spot_MajorAxisLength/spot_MinorAxisLength;
% calculate Aspect Ratio
    fprintf(1,'#%2d %17.2f %11.2f %8.2f\n', k,spot_MajorAxisLength ,
spot_MinorAxisLength, Aspect_ratio);
    % Put the "blob number" labels on the "boundaries" grayscale image.
    text(spot_centroid(1) + labelShiftX, spot_centroid(2), num2str(k),
'FontSize', 12, 'FontWeight', 'Bold');
end
%-----------------------------------------------------
```
